# Density functional theory insights into the formation mechanisms and reaction rates of Strecker aldehydes

**DOI:** 10.1039/d5ra07604h

**Published:** 2025-11-28

**Authors:** Shota Ishida, Koichi Miyagawa, Mitsuo Shoji

**Affiliations:** a Graduate School of Science and Technology, University of Tsukuba 1-1-1 Tennodai, Tsukuba Ibaraki 305-8571 Japan; b Institute of Livestock and Grassland Science, National Agriculture and Food Research Organization (NARO) 2 Ikenodai Tsukuba Ibaraki 305-0901 Japan ishida.shota342@naro.go.jp; c Center for Computational Sciences, University of Tsukuba 1-1-1 Tennodai, Tsukuba Ibaraki 305-8577 Japan mshoji@ccs.tsukuba.ac.jp

## Abstract

Although Strecker degradation significantly affects food quality, its atomistic-level reaction mechanisms remain poorly understood. In this study, new reaction mechanisms for the initial steps of Strecker degradation leading to the formation of aldehydes or ketones were revealed using a first-principles approach based on density functional theory (DFT). We employed a minimal model consisting of glycine and methylglyoxal, and explored a broad reaction space using the conformational search algorithm, the random sampling method. Our results show that both aldehyde and ketone formation pathways proceed with maximum activation energies of less than 25 kcal mol^−1^. According to rate calculations using the Eyring equation, such activation energies correspond to slow reaction rates at around 298.15 K, but significantly accelerated rates at elevated temperatures above 373 K. We also demonstrated that solvation effects influence the preference between two distinct reaction pathways (Path A and Path B). These newly proposed reaction mechanisms for the initial stage of Strecker degradation provide valuable insights into the promotion or regulations of specific reactions, intermediates and end products during food processing.

## Abstract 2

New reaction mechanisms of the Strecker degradation to form aldehydes are revealed using the theoretical level of density functional theory. Their activation barriers of these reactions are less than 25 kcal mol^−1^.

## Graphical abstract

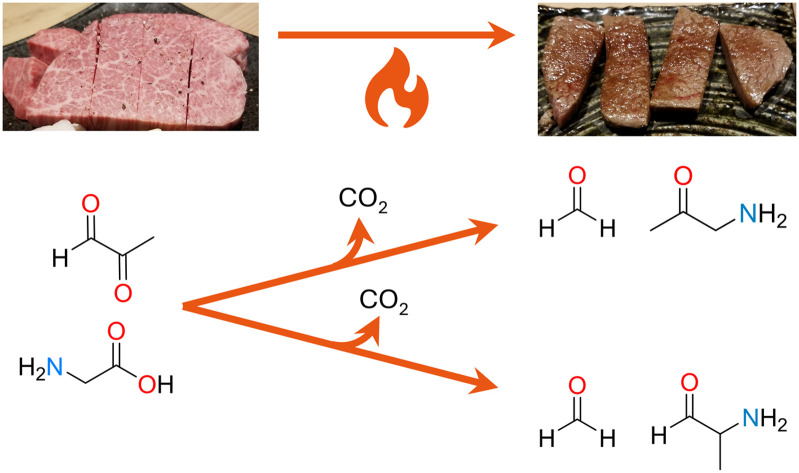

## 1. Introduction

The Maillard reaction, also known as the amino-carbonyl reaction, is a non-enzymatic browning reaction that occurs extensively in food and biological systems.^1^ This reaction is typically divided into three stages.^2^ In the first stage (Stage I), carbonyl groups of reducing sugars condense with amino groups of amino acids to form Schiff bases, which then undergo Amadori rearrangement to yield Amadori compounds. In the second stage (Stage II), these Amadori compounds undergo dehydration and degradation reactions, resulting in the formation of various carbonyl compounds, particularly α-dicarbonyl compounds. In the third stage (Stage III), the highly reactive intermediates formed in Stage II, such as α-dicarbonyl compounds, react further with amino acids or polymerize to form complex high-molecular-weight compounds, including melanoidins, along with flavour components. The Maillard reaction proceeds through a series of chemical intermediates. Among these, Strecker degradation occurs during the State II due to reactions between the substrates and intermediate products of the Maillard reaction.^3,4^ This degradation involves the loss of a carbon atom and leads to aldehyde or ketone formation.^5,6^ Because both the Maillard reaction and Strecker degradation play critical roles in determining food quality, many experimental studies have been conducted.^7–9^ However, the detailed reaction mechanisms remain insufficiently understood. One major difficulty lies in identifying the numerous transient intermediates and their mutual interactions. Additionally, many factors influence the kinetics of these reactions, including pH, temperature, reaction time, water activity, substrate reactivities and reactant concentrations.^10^ In fact, a reaction scheme for Strecker degradation leading to pyrazine formation was proposed by Schönberg and Moubacher^5^ and further elaborated by Rizzi.^11^ However, most of the intermediates proposed in their mechanisms have not yet been detected experimentally.

**Fig. 1:**
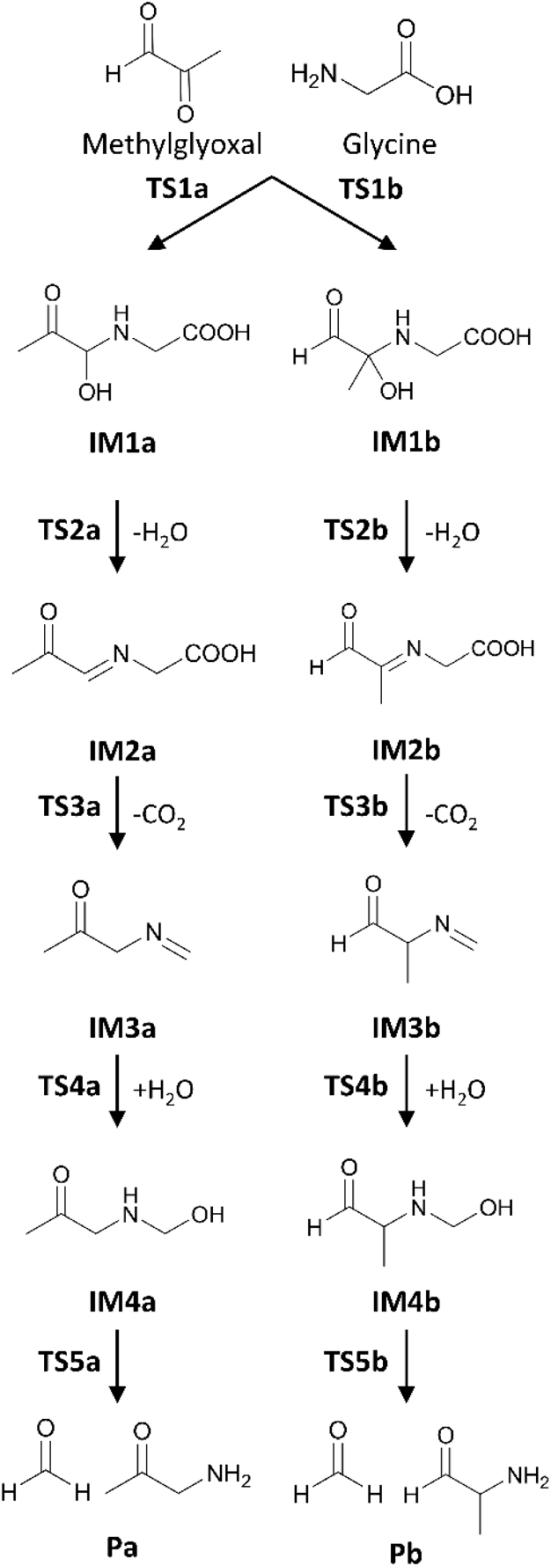
Reaction schemes of the Strecker degradation proposed by Rizzi^11^ and Gao *et al.*^23^

Computational chemistry has been employed to study complex chemical reactions, and it provides precise information about molecular structures and their stabilities. The Maillard reaction has been investigated in a number of computational studies to date.^12–23^ In particular, Shipar and Jalbout^15^ examined the Strecker degradation pathway from methylglyoxal (MGO) and glycine (Gly) to pyrazine, evaluating the Gibbs free energies of the intermediate states (IMs).^15,18^ They focused on the ionization states of Gly, concluding that the deprotonated Gly in aqueous solution was the most favourable condition for pyrazine formation. However, their results were insufficient to fully elucidate a favourable reaction pathway, as their energy profiles were evaluated based solely on intermediate states (IMs) without identifying the corresponding transition states (TSs). Gao *et al.*^23^ extended this investigation by evaluating the relative energies of both IMs and TSs in the Maillard reaction. Fig. 1 shows the Strecker degradation scheme proposed by Rizzi^11^ and Gao *et al.*^23^ Among these reaction pathways, the steps involving carbon dioxide desorption, indicated as TS3a and TS3b in Fig. 1, exhibit very high activation barriers of approximately 50 kcal mol^−1^. Additionally, Ren *et al.*^22^ reported that the carbon dioxide desorption is the rate-determining step of the Strecker degradation, with an activation energy of 66.3 kcal mol^−1^. Such a high activation energy reaction is unlike to proceed at temperatures between 100 °C and 140 °C, where Maillard reaction products are commonly observed.^7,8,24–27^ Therefore, alternative reaction pathways with lower energy barriers are likely to exist.

In our previous studies, we demonstrated that the most stable IMs can be efficiently identified using our developed method, the random sampling (RS) method. In the RS method, three different levels of theory-molecular mechanics, semiempirical methods, and first-principles methods are employed to accelerate the global conformational search, starting from randomly distributed initial atomic geometries.^28,29^

In the present study, the RS method was applied to determine the most stable TSs and the most feasible reaction pathways. Reaction rates and the temperatures required for these reactions to proceed were evaluated based on Gibbs free energy profiles along the Strecker degradation pathway. Additionally, the solvation effect from water molecules was investigated by applying the polarized continuum model (PCM), and the results were compared to those obtained under gas-phase conditions.

## 2. Materials and methods

### 2.1. Computational methods

MGO, Gly, and two water molecules were included as reactants in the Strecker degradation model, and the total number of atoms was kept constant. The water molecules play a crucial role in facilitating proton transfers during the reactions. These molecules represent the simplest yet essential species, which is important for minimizing computational cost. Density functional theory (DFT) calculations were performed at the M06-2X/6-311G* level of theory. This method has been previously applied to investigate the kinetic parameters of the Maillard reaction.^23^ All structures of reactant (R), product (P), IMs and TSs were fully optimized geometrically, followed by frequency calculations. We confirmed that all TSs possess a single imaginary frequency, whereas IMs exhibit none. The Gibbs free energies were calculated by

1 *G* = *H* − *TS*

where *H*, *T* and *S* are the enthalpy, temperature and entropy, respectively. The *H* is given by

2 *H* = *U* + *RT*

where *U* is the internal energy which includes (i) the electronic total energy, (ii) zero-point vibrational energy correction, (iii) thermal correction contributions from vibration, rotation and translation, and *R* is the gas constant. The *S* is given by

3 *S* = *S*_el_ + *S*_vib_ + *S*_rot_ + *S*_trans_

where *S*_el_ is electronic entropy, *S*_vib_ is vibrational entropy, *S*_rot_ is rotational entropy, and *S*_trans_ is the translational entropy.

The calculations were carried out separately for two phases – the gas phase and the aqueous phase – to simulate two extreme environmental conditions, as actual reactions occurring on food surfaces correspond to an intermediate environment between these two phases.^23^ For the aqueous-phase calculations, the polarized continuum model (PCM) with the dielectric constant of water (*ε* = 78.39) was employed. All geometry optimization and frequency calculations were performed using the Gaussian 16 program package (revision C.01).^30^ Molecular structures were visualized using GaussView 6.^31^ To confirm the absence of alternative TSs, the Nudged Elastic Band (NEB) method implemented in the NWChem 7.0.2 program package was used.^32,33^

The investigated reaction pathways are presented in Fig. 1. The molecular structures of these intermediate states were primarily based on the studies by Shipar^15^ and Gao *et al.*^23^ As shown in Fig. 1, two different isomers (IM1a and IM1b) are formed depending on the binding position on MGO. Both reaction routes, namely, the substituted aldehyde pathway (Path A) and the substituted ketone pathway (Path B), were investigated in the present study. For some reaction steps exhibiting high energy barriers, alternative pathways with lower energies were searched using the RS algorithm.^28,29^ This RS method was employed to search for stable conformations in both intermediate and transition states. For each state, at least 100 initial geometries were generated and evaluated using the RS method.

### 2.2. Reaction rate

The reaction rate constants (*k*) were evaluated using Eyring's equation.^34^4
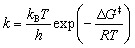
where *k*_B_ is Boltzmann's constant, *h* is Planck's constant. Reaction temperatures were set at 298.15, 373.15, and 393.15 K. These temperatures are commonly used during heating processes that produce aroma compounds *via* the Maillard reaction.^7–9,24–27^ The change in Gibbs free energy from the reactant, Δ*G*, is given by

5 Δ*G* = *G* − *G*(R)

where *G*(R) are Gibbs free energy in reactant. The activation barrier for each reaction step is denoted as Δ*G*^‡^.

## 3. Results and discussion

### 3.1. Reaction mechanism of Strecker degradation

**Fig. 2:**
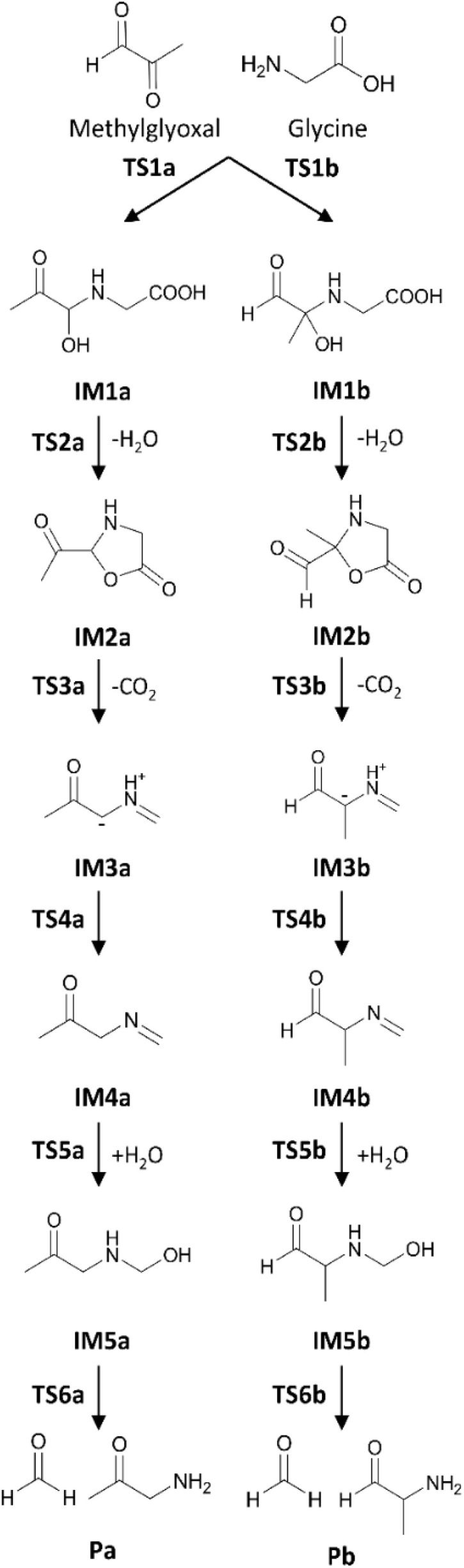
Reaction schemes of the Strecker degradation revealed in the present study.

By exploring alternative reaction pathways, particularly to reduce the highest activation barrier associated with carbon dioxide desorption shown in Fig. 1 (TS3a and TS3b), we discovered new pathways involving a 5-oxooxazolidine species. These pathways are preferable in terms of stability and reactivity. Subsequently, we investigated the complete reaction pathways leading to the formation of aldehyde and ketone products. The new reaction schemes are summarized in Fig. 2. The following sections provide a detailed description of IMs and TSs involved in these reactions. The temperature used for free energy (Δ*G*) calculations in this section was set at *T* = 298.15 K. Their influence on reaction rates at different temperatures is discussed in Section 3.2.

**Fig. 3:**
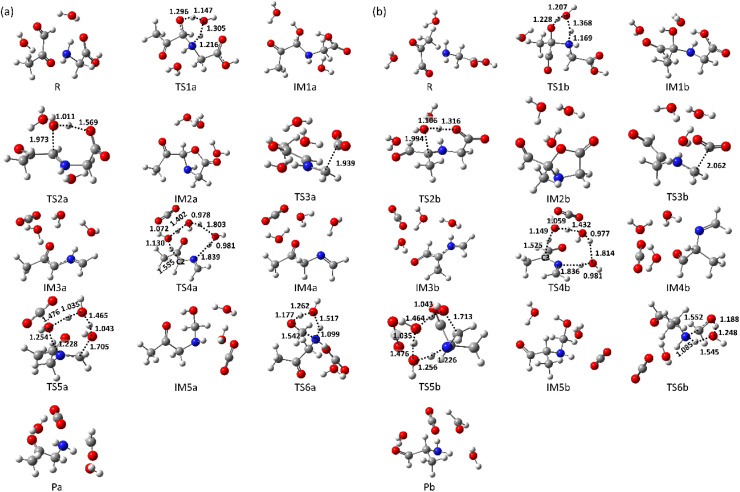
Optimized geometries in the gas phase calculated at the M06-2X/6-311G(d) level of theory. (a) States along the aldehyde-substituted pathway (Path A) and (b) states along the ketone-substituted pathway (Path B). Key atomic distances are given in angstroms (Å).

The optimized molecular structures of all states, R, IMs, TSs, and P, in the gas phase are shown in Fig. 3. In the first step (R → IM1s), MGO and Gly approach each other, and MGO reacts with the amino group of Gly to form the complexes IM1a and IM1b, with relative formation energies of Δ*G*(IM1a) = −9.9 kcal mol^−1^ and Δ*G*(IM1b) = −5.1 kcal mol^−1^, respectively. This reaction requires a proton transfer from the amine group to the aldehyde or ketone group concomitant with C–N bond formation, resulting in the formation of a characteristic six-membered ring in TSs (TS1a and TS1b). The calculated relative free energies of these TSs are Δ*G*(TS1a) = 11.2 kcal mol^−1^ and Δ*G*(TS1b) = 14.3 kcal mol^−1^. As shown in Fig. 3, the bond lengths within the six-membered rings are very similar between TS1a and TS1b. These results indicate that variation in the MGO binding site has negligible effects on this initial reaction step. These reactions (R → [TS1a] → IM1a and R → [TS1b] → IM1b) are fully consistent with previous theoretical results reported by Shipar^15^ and Gao *et al.*^23^ Notably, IM1a was experimentally detected in the reaction of Gly and MGO at pH 7.0 and 37 °C after 3 hours.^35^ The formation of IM1a at low temperature is reasonable given the low energy barrier of TS1a (Table S2).

Next, IM2 is formed *via* the intermolecular dehydration of IM1 (IM1 → IM2). In the corresponding TS (TS2), the hydroxy group is protonated by the carboxyl group and subsequently eliminated as a water molecule. During this reaction, a seven-membered ring is transiently formed to facilitate the proton transfer from the carboxyl group to the hydroxy group. The calculated relative free energies for TS2a and TS2b are Δ*G*(TS2a) = 14.5 kcal mol^−1^ and Δ*G*(TS2b) = 15.8 kcal mol^−1^, respectively. Among the IM2 species, the structures containing a five-membered ring are particularly stable, with the relative free energy of Δ*G*(IM2a) = −9.0 kcal mol^−1^ and Δ*G*(IM2b) = −7.7 kcal mol^−1^. Formation of the five-membered ring reduces the number of unsaturated bonds remaining after dehydration, contributing to the increased stability of the IM2 structures. Compared to previous result reported by Gao *et al.*,^23^ the main difference lies in the position of the deprotonated hydrogen atom. Although the energy difference at the TS is relatively small (ΔΔ*E*(TS2a) = −0.8 kcal mol^−1^), the 5-oxooxazolidine species (IM2a) is significantly more stable than the previously reported IM2a, with an energy difference of ΔΔ*E*(IM2a) = 4.9 kcal mol^−1^.

Desorption of carbon dioxide from the carboxyl group of IM2 yields IM3 (IM2 → IM3). The calculated relative free energies for the TS3s are Δ*G*(TS3a) = 7.9 kcal mol^−1^ and Δ*G*(TS3b) = 8.9 kcal mol^−1^. These TS3s are more stable than the corresponding TS2s, indicating a lower energy barrier for CO_2_ release. The resulting IM3s are similarly stabilized with the relative free energies of Δ*G*(IM3a) = −8.5 kcal mol^−1^ and Δ*G*(IM3b) = −8.5 kcal mol^−1^, comparable to those of IM2s. In the reaction scheme proposed by Shipar^15^ and Gao *et al.*,^23^ CO_2_ desorption was assumed to occurred from the protonated carboxyl group, requiring a simultaneous proton transfer. While an alternative pathway involving proton transfer prior to CO_2_ desorption is conceivable, our calculations show that such proton-transferred state is highly unstable. Therefore, the previously proposed IM2a in Fig. 1 is a non-reactive species.

The next step involves the formation of IM4 from IM3, in which a proton in the imine group is relayed to C2 in IM3a or C3 in IM3b *via* surrounding three water molecules (IM3 → IM4). The calculated relative free energies for the TS4s are Δ*G*(TS4a) = 5.7 kcal mol^−1^ and Δ*G*(TS4b) = 10.7 kcal mol^−1^. A substantial difference is observed between the energy barriers of TS4a and TS4b, suggesting differing reactivity between two pathways. The NBO charge on C2 atom in IM3a is −0.25, whereas that on C3 in IM3b is −0.03. The difference in energy barriers between TS4a and TS4b can be attributed to the differing basicities of the proton acceptor sites in IM3a and IM3b. The resulting IM4s are more stabilized than the corresponding IM3s, with relative energies of Δ*G*(IM4a) = −16.3 kcal mol^−1^ and Δ*G*(IM4b) = −12.5 kcal mol^−1^.

The next step involves formation of IM5 from IM4 through hydrolysis followed by proton transfer (IM4 → IM5). The calculated relative free energies of the TS5s are Δ*G*(TS5a) = −0.7 kcal mol^−1^ and Δ*G*(TS5b) = 7.0 kcal mol^−1^. Notably, proton transfer *via* a single water molecule results in a high energy barrier, especially in TS5b (Δ*G*(TS5b′) = 12.4 kcal mol^−1^), where the relative free energy reaches Δ*G*^‡^(TS5b′) = 24.9 kcal mol^−1^. Therefore, two water molecules are essential to facilitate proton transfer to the imine group with a lower energy barrier. The resulting IM5s are highly stabilized with relative free energies of Δ*G*(IM5a) = −27.4 kcal mol^−1^ and Δ*G*(IM5b) = −22.7 kcal mol^−1^.

The final step involves the formation of P from IM5 *via* the dissociation of formaldehyde. The calculated free energies for the TS6s are Δ*G*(TS6a) = −4.0 kcal mol^−1^ and Δ*G*(TS6b) = 0.1 kcal mol^−1^, while the P states are Δ*G*(Pa) = −12.9 kcal mol^−1^ and Δ*G*(Pb) = −4.1 kcal mol^−1^. In this step, proton transfer proceeds *via* a single water molecule.

**Fig. 4:**
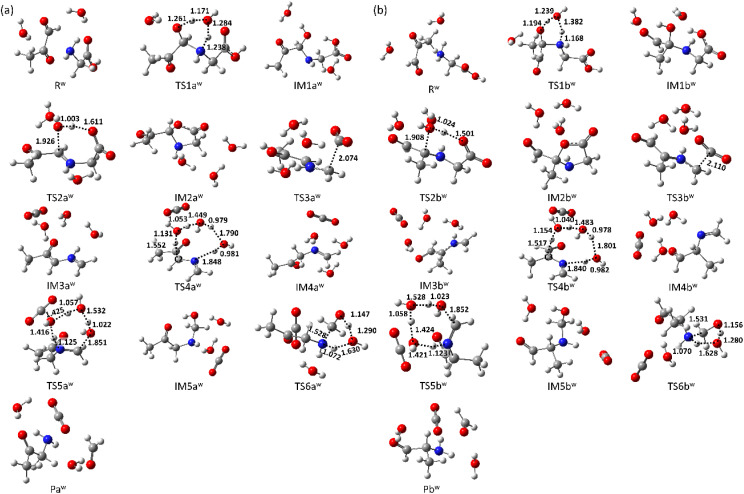
Optimized geometries in the aqueous phase calculated at the M06-2X/6-311G(d) level of theory. (a) States along the aldehyde-substituted pathway (Path A) and (b) states along the ketone-substituted pathway (Path B). Key atomic distances are given in Å.

The optimized structures of all calculated states in aqueous phase are shown in Fig. 4. These geometries are nearly identical to those obtained in the gas phase calculations. These results suggest that the overall reaction mechanism is not significantly affected by the phase (gas *vs.* aqueous).

**Fig. 5:**
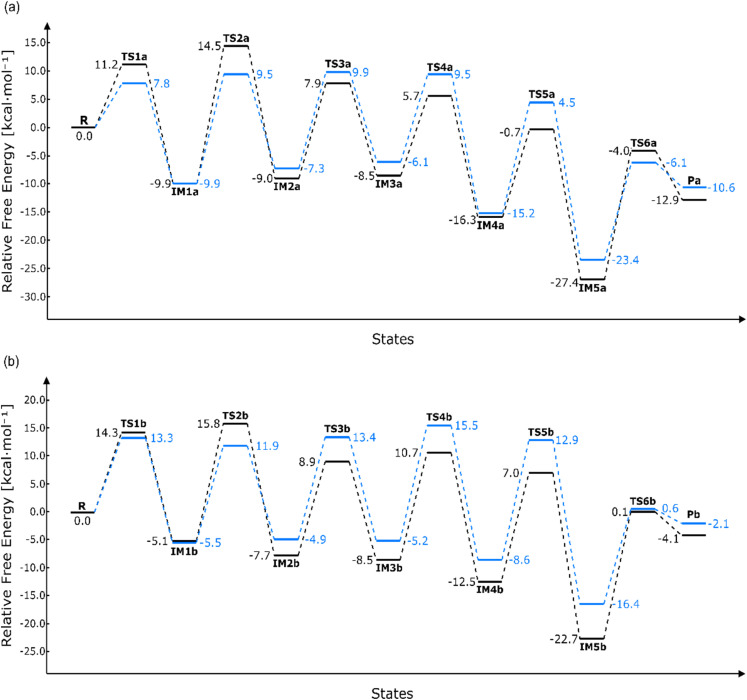
Calculated Gibbs free energy diagrams for the route of aldehyde substituted (a) and the ketone substituted (b) at *T* = 298.15 K. Results in the gas phase and water phase are coloured black and blue, respectively.

The energy diagrams of the Strecker degradation pathways are summarized in Fig. 5. All activation energy barriers (Δ*G*^‡^) for each reaction step are provided in the SI (Table S2). Among all the energy barriers, the steps with highest activation energy barriers are IM1a-TS2a (Δ*G*^‡^(TS2a) = 24.4 kcal mol^−1^), IM5b-TS6b (Δ*G*^‡^(TS6b) = 22.8 kcal mol^−1^), and IM1a^W^-TS3a^W^ (Δ*G*^‡^(TS3a^W^) = 19.8 kcal mol^−1^) and IM4b^W^-TS5b^W^(Δ*G*^‡^(TS5b^w^) = 21.5 kcal mol^−1^). The values in parenthesis represent the required free energy changes at *T* = 298.15 K. Notably, these activation energies are significantly lower than the previously reported value ofΔ*E*^‡^(TS2a in Fig. 1) = 58 kcal mol^−1^.^23^ The current high activation energy barrier steps, TS2 and TS6, are decomposition reactions mediated by a single water molecule. Therefore, these steps are sensitive to the local solvation environment.

The activation energies of Path A and Path B in both the gas phase and aqueous phase are summarized in Table 1. These activation energies are lower than previous reports for the Maillard reaction. Brands and Boekel^24^ reported that the activation energy for the reaction between d-glucose and the lysine binding protein was 128 kJ (=30.6 kcal mol^−1^). Ayranci and Dalgıç^36^ reported that the activation energies for reactions between various monosaccharaides and lysine range from 116.6 to 162.5 kJ (=27.87–38.84 kcal mol^−1^). These results suggest that the decomposition of monosaccharides into MGO and the type of amino acid involved are also influencing factors for the overall activation energies in the Maillard reaction.

**Table 1:** Calculated reaction rate constants for the reactions of the Strecker degradation at different temperatures (*T*)

*T* [K]	Path	Δ*G*^‡^ [kcal mol^−1^]			Reaction rate [s^−1^]		
		298.15	373.15	393.15	298.15	373.15	393.15
Gas phase	A	24.4	24.8	24.9	8.09 × 10^−6^	2.32 × 10^−2^	1.18 × 10^−1^
	B	22.8	23.1	23.2	1.20 × 10^−4^	2.30 × 10^−1^	1.04
Aqueous phase	A	19.7	20.9	21.3	2.25 × 10^−2^	4.47	11.8
	B	21.5	22.6	23.0	1.08 × 10^−3^	4.51 × 10^−1^	1.34

### 3.2. Reaction rate of Strecker degradation

The reaction rate constants of Path A and Path B in the gas phase and aqueous phase at temperatures of *T* = 298.15, 373.15, and 393.15 K are shown in Table 1. The reaction rates for both pathways are sufficiently fast to proceed at *T* = 373.15 K or higher in both phases. These results are in good agreement with experimental observations that Maillard reaction products are formed at temperatures above *T* = 373.15 K.^7^ When comparing the contributions of reaction rates at *T* = 298.15 K and 373.15 K in Table 1, the rate increased in *T*, 373.15/298.15 = 1.25 is higher than the increased inΔ*G*^‡^, 24.8/24.4 = 1.016. This suggests that temperature is one of the primary factors contributing to the activation of the reaction.

The reaction rate values of both pathways are relatively low at *T* = 298.15 K in both phases. Although the Strecker degradation is typically observed above *T* = 373.15 K, several studies have reported that the Strecker degradation proceeded even at below *T* = 373.15 K. For example, Wietstock *et al.*^37^ detected Strecker aldehydes in beer stored at 28 °C (=301.15 K) for 12 weeks. Gibson *et al.*^38^ reported that the supplementation of isoleucine with fresh beer and subsequent aging at 37 °C (=310.15 K) for 7 days promoted the formation of 2-metylbutanal, concluding that Strecker degradation increased aldehyde concentration. Xing and Yaylayan^3^ detected Strecker aldehydes from the reaction between MGO and various amino acids through ball milling at temperatures between 25–38 °C (=298.15–311.15 K) for 30 minutes. This reaction was able to occur at low temperatures and within a short time, and many kinds of Strecker aldehydes, including formaldehyde, were not detected. These results suggest that the production of Strecker aldehyde is limited under low temperature and short-duration conditions. Our findings indicate that the reaction rate in the aqueous phase tend to be slightly higher than in the gas phase. This suggests that water solvent enhances the Strecker degradation during low-temperature fermentation processes such as beer aging. In contrast, under high-temperature conditions above 100 °C-such as during cooking-the solvent effect may be minimal or negligible as the temperature exceeded the boiling point of water.

## 4. Conclusions

The reaction mechanisms of the Strecker degradation from an amino acid (Gly) and an α-dicarbonyl compound (MGO) leading to aldehyde formation were investigated using DFT. Two distinct reaction pathways were identified, based on the condensation of Gly with either the aldehyde or ketone functional group of MGO. The highest activation barriers were found to be Δ*G*^‡^ = 23–25 kcal mol^−1^ in the gas phase and Δ*G*^‡^ = 19–23 kcal mol^−1^ in the aqueous phase. These barriers correspond to reaction rate constants of *k* = 10^−2^–10^−6^ s^−1^ at *T* = 298.15 K, *k* = 10^−2^–1.0 s^−1^ at *T* = 373.15 K, and *k* = 10–10^−1^ s^−1^ at *T* = 393.15 K, respectively. These findings indicate that the reactions proceed slowly at low temperatures, *e.g. T* = 298.15 K, but become significantly faster at elevated temperatures above *T* = 373.15 K. The rate-determining steps in both pathways involve water-related processes—either decomposition or proton transfers—which are sensitive to the solvation effects. Our results suggest that reactions in the aqueous phase are generally faster than those in the gas phase. Additionally, Path A is more favourable than Path B in the aqueous phase, whereas Path B proceeds more readily than Path A in the gas phase. These new insights into the mechanisms and kinetics of the initial stage of the Strecker degradation provide a fundamental basis for understanding food processing. They also offer potential for targeted regulation of specific reactions and intermediates to control the formation of flavour compounds and other products in food systems.

## Author contributions

Shota Ishida: writing – original draft, methodology, investigation, formal analysis, data curation, conceptualization. Koichi Miyagawa: writing – review & editing, methodology, investigation, formal analysis. Mitsuo Shoji: writing – review & editing, supervision, methodology, investigation, formal analysis.

## Conflicts of interest

The authors declare no conflicts of interests.

## Supplementary Material

RA-015-D5RA07604H-s001

## Data Availability

The data supporting this article have been included as part of the supporting information (SI). Supplementary information (SI): (1) DFT precision check against a coupled cluster method, (2) rate constants for all transition states and (3) the optimized structures of all states in *XYZ* format. See DOI: https://doi.org/10.1039/d5ra07604h.
