# Supplementary material for: Density functional theory insights into the formation mechanisms and reaction rates of Strecker aldehydes
Source: RSC Adv. 2025 Nov 28;15(55):46959–66. doi: 10.1039/d5ra07604h (PMC12661492; doi:10.1039/d5ra07604h)
Supplement: RA-015-D5RA07604H-s001 [file RA-015-D5RA07604H-s001.pdf]

Supplementary Information (SI) for RSC Advances.
This journal is © The Royal Society of Chemistry 2025

Supplementary information

Density functional theory insights into the formation mechanisms and reaction rates of Strecker aldehydes

Shota Ishida [a, b, *], Koichi Miyagawa [c], Mitsuo Shoji [c, *]

[a] Graduate School of Pure and Applied Sciences, University of Tsukuba, 1-1-1 Tennodai, Tsukuba, Ibaraki 305-8571, Japan.

[b] Institute of Livestock and Grassland Science, National Agriculture and Food Research Organization (NARO), Tsukuba, Japan.

[c] Center for Computational Sciences, University of Tsukuba, 1-1-1 Tennodai, Tsukuba, Ibaraki 305-8577, Japan.

* Corresponding authors: Shota Ishida (ishida.shota342@naro.go.jp)

and Mitsuo Shoji (mshoji@ccs.tsukuba.ac.jp)

Table of Contents

Verification of the accuracy of theoretical levels

Table S1 Correlation factors of various theoretical methods

Figure S1. Scatter plot of the M06-2X calculations

Figure S2. Scatter plot of the B3LYP calculations

Figure S3. Scatter plot of the HF calculations

Table S2. Energy barriers for each transition state

Table S3. Thermal parameters of rate-limiting steps

Atomic coordinates optimized at the M06-2X/6-311G* in the gas phase and water phase are provided in the XYZ file format

Verification of the accuracy of theoretical levels

Twenty-five molecular structures–comprising 13 intermediates and 12 transition states along two different paths (Paths A and B of Figure 2)–were used to assess the accuracy of the DFT method. DFT functionals B3LYP[S1] and M06-2X[S2], combined with basis sets 6-31G, 6-311G and 6-311G(d)[S3,S4] were tested and compared against a reliable coupled cluster method, DLPNO-CCSD(T_1)/aug-ccpVTZ[S5,S6]. Grimme's dispersion (vdW) correction[S7] was also evaluated. The DLPNO-CCSD(T_1)/aug-ccpVTZ[S8,S9] calculations were performed using the ORCA program package [S10,S11], while all other calculations were carried out using Gaussian 16 (revision C.01) program package[S12].

The total energies (E_i^{CC}, E_i^{X}) = (x, y) are fitted to least squares lines

$$y_{LSL} = C_0 + C_1 x \qquad \text{(S1)}$$

, where E^X is the energy computed with theoretical method X (e.g. X = CC, DFT…), and C_0 and C_1 are the fitted offset and slope coefficients for method X, respectively. The reciprocal $1/C_1$ corresponds to the scale factor for energies in method X. In other words, a method with $C_1 < 1$ underestimates energies, whereas a method with $C_1 > 1$ overestimates them.

The correlation coefficient for the DLPNO-CCSD(T_1) results is quantified by the coefficient R value, defined as:

$$R = \frac{\sum_i \left(E_i^X - \overline{E^X}\right)\left(E_i^{CC} - \overline{E^{CC}}\right)}{\sqrt{\sum_i \left(E_i^X - \overline{E^X}\right)^2}\sqrt{\sum_i \left(E_i^{CC} - \overline{E^{CC}}\right)^2}} \qquad \text{(S2a)}$$

$$\overline{E^X} = \frac{1}{N}\sum_i E_i^X \qquad \text{(S2b)}$$

, where $\overline{E^X}$ is the average energy. If the R value is closer to 1, the method exhibits stronger correlation with the CC method, indicating that its prevision is more comparable to that of the CC method.

In Table S1, the coefficients of the linear fits and their corresponding R values are summarized. All correlation plots for energy comparisons are shown in Figures S1, S2, S3. Among the theoretical levels evaluated, the R values for M06-2X/6-311G(d), B3LYP/6-311G(d), and HF/6-311G(d) are ranked in the following order:

M06-2X (R=0.976) > HF (R=0.928) > B3LYP (R=0.902).

Based on the C_1 values in Table S1, C_1=0.982 of M06-2X/6-311G(d) is closest to 1.0 among the theoretical levels tested. These results suggest that the M06-2X functional performs the best. We also find that B3LYP exhibits lower precision than HF. The application of Grimme's D3 correction was not found to be ineffective–or even detrimental for calculations using B3LYP/6-311G(d). Therefore, for the states in the SD, the van der Waals correction is necessary when using DFT functionals.

Regarding basis set dependence, the R values for M06-2X follow the same ordering:

6-311G(d)(R=0.976) > 6-311G(R=0.770) > 6-31G(R=0.747).

More precisely, improvements in *R* are observed with each expansion of the basis set in M06-2X method,

[6-311G -> 6-311G(d)] (ΔR=0.206) > [6-31G -> 6-311G] (ΔR=0.023).

Similarly, the B3LYP methods show the same order as:

[6-311G->6-311G(d)](ΔR=0.135) > [6-31G -> 6-311G] (ΔR=0.012).

Accordingly, the 6-311G(d) basis set is recommended for use.

Table S1 shows the total times required for the single point calculations of all 25 states. The times relative to the execution time of HF/6-31G (157 sec.= 2.6 min.) are given as relative time ratios (r_t) in parenthesis in Table S1. The r_t values are largely insensitive to the functionals: r_t=1.78 (M06-2X/6-311G(d)), r_t=1.53 (B3LYP/6-311G(d) and r_t=1.90 (HF/6-311G(d)). These depend more strongly on the size of the basis set.

The total computational times increases in the following order:

r_t=1.17 (M06-2X/6-31G) < r_t=1.45 (M06-2X/6-311G) < r_t=1.81 (M06-2X/6-311G(d))

M06-2X/6-311G(d) represents a moderate computational cost.

Thus, M06-2X/6-311G(d) emerges as an appropriate method that balances accuracy with computational expense.

References

S1 Y. Zhao, D. G. Truhlar, Theor. Chem. Acc., 2008, 120, 215-241.
S2 A. D. Becke, J. Chem. Phys. 1993, 98(1372), 5648.
S3 T. Clark, J. Chandrasekhar, G. W. Spitznagel, P.V.R. Schleyer, J. Comput. Chem. 1983, 4, 294-301.
S4 R. Krishnan, J. S. Binkley, R. Seeger, J. A. Pople, J. Chem. Phys. 1980, 72, 650-654.
S5 J. Sponer, K. E. Riley, P. Hobza, PCCP, 2008, 10, 2595-2610.
S6 J. Sponer, J. E. Sponer, A. Mladek, P. Banas, P. Jurecka, M. Otyepka, Methods, 2013, 64, 3-11.
S7 S. Grimme, J. Antony, S. Ehrlich, H. Krieg, J. Chem. Phys. 2010, 132, 154104.
S8 Y. Guo, C. Riplinger, U. Becker et al., J. Chem. Phys. 2018,148, 011101.
S9 R. A. Kendall, T. H. Dunning, Jr., R. J. Harrison, J. Chem. Phys. 1992, 96, 6796.
S10 F. Neese, The ORCA program system Wiley Interdiscip. Rev.: Comput. Mol. Sci. 2012, 2(1), 73-78.
S11 F. Neese, Wiley Interdiscip. Rev.: Comput. Mol. Sci., 2022, 12,1, e1606.
S12 M. J. Frisch, G.W. Trucks, H.B.Schlegel, et al., Gaussian16 (Revision C.01), Gaussian Inc., Wallingford CT, 2016.

Table S1. Correlation factors *R* for relative energies of various theoretical methods, relative to DLPNO-CCSD(T_1)/aug-ccpVTZ

Method	C_0 / a.u.	C_1	R	Time/sec. (ratio (r_t))
M06-2X/6-31G	-224.669	0.681	0.747	185 (1.17)
M06-2X/6-311G	-208.393	0.704	0.770	229 (1.45)
M06-2X/6-311G(d)	-13.375	0.982	0.976	285 (1.81)
M06-2X-D3/6-311G(d)	-13.351	0.982	0.976	280 (1.78)
B3LYP/6-31G	-276.803	0.607	0.755	157 (1.00)
B3LYP/6-311G	-261.162	0.630	0.767	190 (1.21)
B3LYP/6-311G(d)	-74.830	0.895	0.902	241 (1.53)
B3LYP-D3/6-311G(d)	-96.755	0.864	0.892	263 (1.67)
HF/6-31G	181.140	1.252	0.941	157 (1)
HF/6-311G	206.760	1.289	0.937	257 (1.63)
HF/6-311G(d)	430.256	1.607	0.928	299 (1.90)
DLPNO-CCSD(T_1)/aug-ccpVTZ	0	1	1	238578 (1519.60)

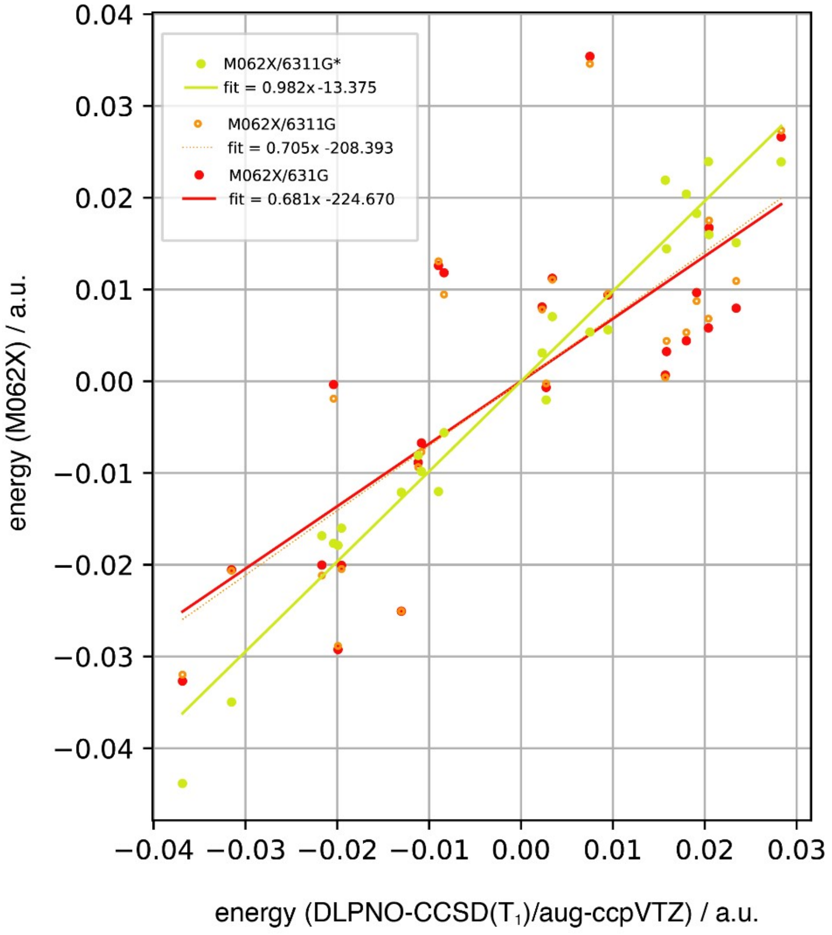

Figure S1. Scatter plot of energies calculated by M06-2X against reference results from DLPNO-CCSD(T_1)/aug-ccpVTZ.

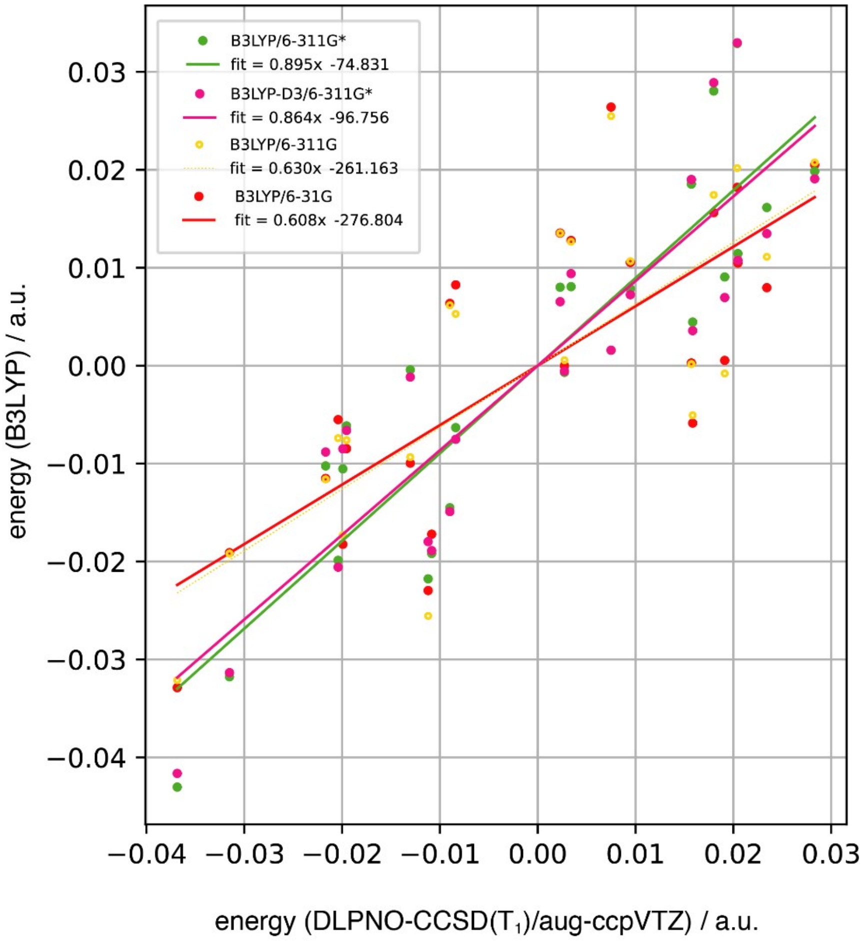

Figure S2. Scatter plot of energies calculated by B3LYP against reference results from DLPNO-CCSD(T_1)/aug-ccpVTZ.

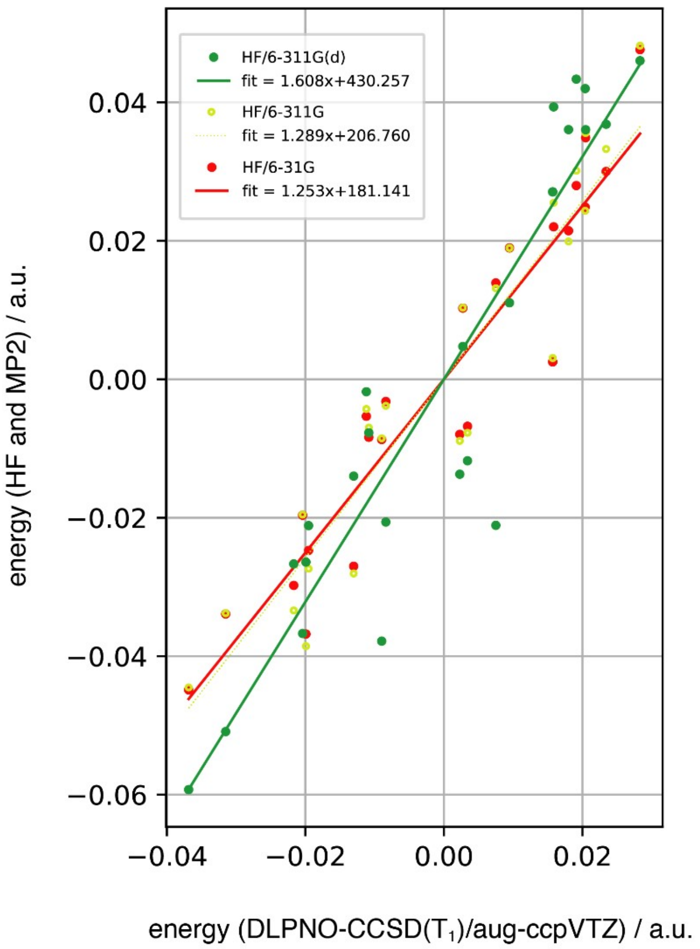

Figure S3. Scatter plot of energies calculated by HF against reference results from DLPNO-CCSD(T_1)/aug-ccpVTZ.

Table S2 Energy barriers for each transition state

		G(TS) [kcal / mol]	Ground state (GS)	G(GS) [kcal / mol]	$\Delta G^{\ddagger}$ [kcal / mol]
Gas phase	TS1a	11.2	R	0	11.2
	TS2a	14.5	IM1a	-9.9	**24.4**
	TS3a	7.9	IM1a	-9.9	17.8
	TS4a	5.7	IM1a	-9.9	15.6
	TS5a	-0.7	IM4a	-16.3	15.6
	TS6a	-4.0	IM5a	-27.4	23.4
	TS1b	14.3	R	0	14.3
	TS2b	15.8	IM1b	-5.1	20.9
	TS3b	8.9	IM2b	-7.7	16.6
	TS4b	10.7	IM3b	-8.5	19.2
	TS5b	7.0	IM4b	-12.5	19.5
	TS6b	0.1	IM5b	-22.7	**22.8**
Water phase	TS1a	7.8	R	0	7.8
	TS2a	9.5	IM1a	-9.9	19.4
	TS3a	9.9	IM1a	-9.9	**19.8**
	TS4a	9.5	IM1a	-9.9	19.4
	TS5a	4.5	IM4a	-15.2	19.7
	TS6a	-6.1	IM5a	-23.4	17.3
	TS1b	13.3	R	0	13.3
	TS2b	11.9	IM1b	-5.5	17.4
	TS3b	13.4	IM1b	-5.5	18.9
	TS4b	15.5	IM1b	-5.5	21.0
	TS5b	12.9	IM4b	-8.6	**21.5**
	TS6b	0.6	IM5b	-16.4	17.0

The values of energy barriers for each transition state were calculated as described in the table.

Underlined values are rate-limiting stage in each pathway.

Table S3 Thermal parameters of rate-limiting steps

		Step	ΔH [kcal mol^{-1}]	ΔS [kcal mol^{-1} K^{-1}]	$\Delta G^{\ddagger}$ [kcal mol^{-1}]		
					T=298.15 K	T=373.15 K	T=393.15 K
Gas	A	IM1-TS2	23.1	-4.45×10^{-3}	24.4	24.8	24.9
phase	B	IM5-TS6	21.3	-4.77×10^{-3}	22.8	23.1	23.2
Water	A	IM4-TS5	14.4	-1.74×10^{-2}	19.7	20.9	21.3
phase	B	IM4-TS5	16.9	-1.53×10^{-2}	21.5	22.6	23.0

The values of energy barriers were calculated by substituting $\Delta G=\Delta H-T\Delta S$ with ΔH: enthalpy changes, T: temperatures, and ΔS: entropy changes, as described in the table.

Atomic coordinates optimized at the M06-2X/6-311G* in the gas phase and water phase are provided in the XYZ file format.

25
Reactants in Path a
C 2.01032500 -0.28962200 -1.76801700
C 1.23951900 -0.50793100 -0.50380300
H 1.69269300 -1.00276500 -2.52746200
O 3.10676600 -1.01840200 1.30001400
H 3.58865000 -0.93064900 2.12516400
C 1.18301000 0.66506900 0.47866200
H 0.73280700 0.39704500 1.44963700
O 0.75148400 -1.56368800 -0.16605600
H 2.51872800 -1.77653100 1.37424900
O 1.56414700 1.77213800 0.21545700
H -0.23643100 2.93858200 0.55826300
H 1.90580600 0.73612800 -2.11562200
H 3.06489400 -0.46447500 -1.53357700
O -1.19330500 2.82529800 0.54102200
H -1.38005500 2.05158800 1.08695100
C -1.83154700 -0.50478200 -1.18631000
C -1.99439800 -0.73199000 0.29461000
O -2.56049200 -1.91253400 0.55270400
O -1.70706100 0.05178800 1.16377000
H -2.65694500 -1.99087200 1.51175600
N -0.94809400 0.61564700 -1.43031900
H -0.88960100 0.81482800 -2.42143900
H -1.24970100 1.46401600 -0.94994600
H -1.40533300 -1.41982600 -1.60464100
H -2.84626000 -0.40052700 -1.59750300

25

TS1a

C 3.03689800 0.14985100 -1.20625300

C 1.56512600 -0.11729200 -1.27801800

H 3.56736900 -0.44507000 -1.94768600

O 1.65326900 -2.38177500 0.34516500

H 2.04227000 -3.14032300 0.78604200

C 0.69812900 0.80726100 -0.41625400

H 0.92074000 0.62592300 0.65413300

O 1.06995500 -0.96406300 -1.99140200

H 1.66916000 -2.54300800 -0.60456200

O 0.85402300 2.07173700 -0.83580800

H -0.25098300 2.66494500 -0.50761900

H 3.20859000 1.21585500 -1.35837500

H 3.38552500 -0.10907300 -0.20277400

O -1.36849400 2.71413300 -0.25463300

H -1.48972400 2.93109300 0.67414400

C -1.13667300 -0.77314800 0.27048300

C -1.45122400 -0.29508200 1.66707000

O -1.79185300 -1.32224300 2.45437900

O -1.41237400 0.84241400 2.04474000

H -1.98166000 -0.97076800 3.33542700

N -0.75901300 0.35896700 -0.57709100

H -0.86568000 0.10124200 -1.55767700

H -1.32356400 1.41513200 -0.36793800

H -0.33512300 -1.51708700 0.32644600

H -2.02548200 -1.26388900 -0.13058300

25

IM1a

C 7.73698300 3.33359300 6.56307600

H 8.46080900 2.63262400 6.97335900

H 7.65077600 4.20231400 7.22124400

H 8.06236500 3.71692200 5.59482600

C 6.40035100 2.65819300 6.44161300

O 6.18701200 1.56361000 6.90665800

C 5.30028800 3.37959300 5.63960200

H 5.41275900 3.03067300 4.60050200

O 4.01384900 3.01310900 6.11128100

N 5.49092300 4.78903100 5.66296800

H 3.82655200 2.06819200 5.92849100

H 5.52175300 5.17252600 6.60685500

C 4.59609300 5.58130600 4.82866700

H 4.23736800 4.96154500 4.00070700

H 5.12390800 6.43534600 4.40416200

C 3.38544800 6.15709500 5.57062400

O 2.68758700 5.30240700 6.32509400

H 3.04615400 4.38843600 6.26440400

O 3.09278200 7.31868900 5.51516500

O 4.01733100 0.37450600 5.72639200

H 4.78994100 0.34710300 6.30765800

H 3.38206200 -0.28356300 6.01412100

O 4.75739500 6.46295200 7.99514500

H 3.86054800 6.12032300 8.08703000

H 4.65243200 7.35466500 7.64814600

25

TS2a

C 7.85678100 2.50189300 6.00391400

H 8.66334400 1.79834300 6.19432300

H 7.93345200 3.35786800 6.67847400

H 7.93330900 2.87271900 4.97794300

C 6.54160600 1.81564400 6.22422500

O 6.42669000 0.64284200 6.48143600

C 5.29643300 2.67102600 6.14933500

H 4.34670200 2.17356000 6.31593400

O 5.49886400 3.05146500 8.07457300

N 5.30602000 3.77302500 5.46190000

H 4.94181400 2.36481000 8.48161500

H 6.18879200 4.24634900 5.22902700

C 4.17390300 4.69646300 5.41137300

H 3.25508700 4.13413900 5.58066200

H 4.15947400 5.17284100 4.43315700

C 4.38804800 5.75896400 6.55112300

O 4.31761700 5.29654700 7.71144700

H 5.01752500 3.94067600 8.07871300

O 4.65252600 6.90402800 6.17468900

O 4.19852800 0.70632400 8.17672500

H 4.87698600 0.23209200 7.67754200

H 3.72004400 0.08501400 8.72829900

O 6.86698800 5.91257100 4.88511900

H 7.71252700 6.34038300 5.02856800

H 6.16720700 6.50599100 5.22721300

25

IM2a

C 7.35246400 2.95518000 5.68021400

H 8.27306600 2.51031100 5.30898900

H 6.87286500 2.28090000 6.39410900

H 7.55846200 3.88659300 6.21420800

C 6.41913600 3.19342000 4.52416600

O 6.63513300 2.84276600 3.39665800

C 5.12740500 3.95901700 4.81995800

H 4.36844500 3.67605200 4.08711500

O 2.96062100 7.26381800 8.33150300

N 5.32389700 5.38986900 4.83191400

H 2.26734200 7.49877500 8.95032000

H 6.20222300 5.57817100 5.31229700

C 4.27123600 5.84682000 5.73488600

H 3.33347600 6.01640200 5.19517400

H 4.54197400 6.75317500 6.27081400

C 4.08335600 4.67988200 6.69258300

O 4.62135500 3.59137500 6.13399400

H 3.00115400 6.29269800 8.26014600

O 3.52718600 4.65488000 7.76005000

O 5.55551500 7.89623200 8.27737700

H 4.59057300 7.79165000 8.40466300

H 5.72477800 8.77144100 7.92515100

O 6.58671100 5.53771600 7.47121000

H 6.54611400 4.92373900 8.20855100

H 6.36072000 6.42319400 7.81783600

25

TS3a

C 3.77003900 -0.53153300 0.28742100

H 4.39830000 0.18697500 0.80826100

H 4.24660000 -0.82832600 -0.65033700

H 3.65940400 -1.43386300 0.89473100

C 2.42204400 0.07727200 0.01644800

O 2.13855100 1.21778400 0.33722500

C 1.43167700 -0.78010700 -0.65406300

H 1.58736200 -1.84169900 -0.79829000

O -0.35397900 0.77316300 1.80522900

N 0.32801100 -0.23374100 -1.07270200

H -0.21959000 -0.18135200 1.84278600

H 0.19515100 0.81108600 -0.95928000

C -0.79836300 -0.87613400 -1.48661300

H -0.64608500 -1.85258800 -1.93663600

H -1.51833900 -0.22352400 -1.96190300

C -1.60616100 -1.50043500 0.16174200

O -0.71695400 -1.91885000 0.84733000

H 0.44816600 1.17117200 1.44548000

O -2.78350000 -1.33426800 0.05268000

O -2.58482100 1.56101300 0.45016300

H -3.09279400 0.77436100 0.22431200

H -1.95930400 1.29674500 1.14904200

O -0.58911300 2.23229400 -1.12148100

H -0.15081200 3.03619100 -0.83644400

H -1.44102600 2.13755300 -0.63159800

25

IM3a

C 8.92595100 4.27296700 7.83439700

H 9.62905200 3.50842600 8.16958200

H 8.75854400 4.94939100 8.67616600

H 9.36665900 4.83056700 7.00774700

C 7.62021300 3.60335000 7.47388500

O 7.05835400 2.88177200 8.32615100

C 7.08527700 3.85439600 6.19820100

H 7.57228900 4.44189200 5.43412800

O 4.03376700 1.65527400 7.04106800

N 5.92302400 3.20194800 5.86044800

H 3.87362800 2.21690100 7.83148800

H 5.51690100 2.57751400 6.58516500

C 5.23150800 3.23105700 4.77393800

H 5.55242500 3.84690500 3.94389600

H 4.33340800 2.63181500 4.74544900

C 6.80126600 3.67261000 10.89723000

O 6.71976900 2.59192800 11.27986700

H 4.14200200 0.75363200 7.35173300

O 6.89411300 4.78241300 10.56916700

O 4.44491700 3.29025500 9.08144500

H 4.45048000 4.22542900 8.81687000

H 5.36180200 2.99971700 8.93725600

O 5.38354700 5.73907100 8.23377000

H 5.94529800 5.44636900 7.50437200

H 5.96177700 5.85324200 8.99549000

25

TS4a

C 0.74728700 2.64588700 -0.72165200

H 0.91811300 2.89270100 -1.77144700

H 1.72619500 2.49132400 -0.26250100

H 0.24650000 3.48359800 -0.23680800

C -0.04272900 1.35533200 -0.66144200

O 0.48298400 0.33408500 -1.14348700

C -1.27645800 1.37587000 0.05375400

H -1.70786800 2.34674700 0.29919400

O -2.03544300 -2.39233600 -0.78347900

N -2.17522300 0.30579300 -0.12549900

H -0.52872900 -2.34795700 0.20584200

H -2.05750400 -1.41166000 -0.77241100

C -3.38294300 0.40789800 0.25182100

H -3.78924900 1.32042300 0.69909200

H -4.04610000 -0.44510300 0.13060400

C 2.77995600 -0.51462000 0.06044400

O 3.10010900 -1.15023500 -0.84088100

H -1.99984700 -2.68325700 -1.69686700

O 2.50908800 0.12256300 0.99359200

O 0.22562000 -1.76881100 0.43306500

H -0.02673000 -0.84713000 1.45852100

H 0.28994400 -1.15085400 -0.33491000

O -0.14500900 0.03997200 2.04917100

H -0.59079300 0.77069600 1.31141300

H 0.74316300 0.33581300 2.28737100

25

IM4a

C 3.82570200 -0.63211000 -0.36434200

C 2.39464400 -0.18070100 -0.22186500

H 4.32493700 -0.04142500 -1.12931300

O 0.13885800 2.77113400 0.28597100

H -2.07183400 1.25713500 -0.98645100

C 1.66508000 -0.64794600 1.03237900

H 2.15972800 -0.17824900 1.89692900

O 1.84777200 0.50041500 -1.05456500

H -0.81485600 2.73093600 0.10777600

O -2.43384100 1.94967800 -0.40376700

H -3.17193400 1.55844000 0.06793500

H 4.36372500 -0.55758200 0.58275200

H 3.83721400 -1.68423100 -0.66354600

O -0.97616100 0.02242000 -1.62699400

H 0.55449400 2.21718100 -0.38352500

C -0.19019100 0.57421100 1.69570400

C -2.07538500 -1.79923100 0.06071000

O -2.72087400 -0.92083500 0.45068100

O -1.46844900 -2.70827100 -0.30569800

H -0.38421300 -0.11595800 -0.86679300

N 0.25785200 -0.33994400 0.94553300

H 1.80458900 -1.73010200 1.12746700

H -0.41236100 0.16294200 -2.39100500

H -1.23164500 0.86503000 1.59980800

H 0.41965500 1.09383400 2.43870000

25

TS5a

C -3.34015600 0.16030400 -0.23536100

C -1.93322400 -0.36899500 -0.29432600

H -3.75738500 0.23790900 -1.23714700

O 1.10361800 -1.63407600 -1.16581000

H 2.55774800 0.30841100 0.34234700

C -1.37879100 -0.94871200 1.00122400

H -1.88656800 -1.91957600 1.13099300

O -1.28695200 -0.32627900 -1.32007500

H 1.91092200 -0.98983200 -1.01795600

O 2.95555200 -0.08871400 -0.52626100

H 3.10900600 0.64592100 -1.12516500

H -3.97635700 -0.45982900 0.39927400

H -3.29838300 1.15708800 0.21369100

O 1.66771700 0.73892800 1.43885500

H 0.33579100 -1.10796800 -1.46534100

C 0.66862100 -2.06177100 0.42608000

C 0.12555600 1.99666200 -0.16092400

O -0.74225300 2.08241300 0.60032800

O 0.92374200 1.95406300 -0.99428800

H 0.78551600 -0.15096700 1.39677500

N 0.04660500 -1.08139000 1.08623000

H -1.73108800 -0.31666400 1.82193700

H 2.01758200 0.91039500 2.31412600

H 1.68456200 -2.26197100 0.75837900

H 0.09899300 -2.95879600 0.18234100

25

IM5a

C 3.80472000 -1.05245200 -0.47158100

C 2.59420000 -0.20645500 -0.16190200

H 4.65800400 -0.41656100 -0.69769400

O 0.28026400 2.16287400 0.61432900

H -1.78599700 0.66432900 -1.62464300

C 1.44560000 -0.91735400 0.54852600

H 1.79990300 -1.13492500 1.56571100

O 2.54926000 0.96433800 -0.46149800

H -1.24946500 2.35543900 -0.32853600

O -2.09281900 2.12854600 -0.76372500

H -2.71085600 1.87722900 -0.07167600

H 4.04067300 -1.72696500 0.35428500

H 3.58555900 -1.67560300 -1.34354000

O -1.46971100 -0.24725200 -1.79850000

H 1.12026200 2.05333500 0.14242600

C 0.05817400 0.96927400 1.35510100

C -2.33635800 -1.47442800 0.36824500

O -2.80794400 -0.46267600 0.67984300

O -1.91842200 -2.51367000 0.09694500

H -0.27348700 -0.19155600 -0.31003400

N 0.17478500 -0.24827200 0.60560600

H 1.30710900 -1.89881000 0.08112300

H -1.22119400 -0.32062000 -2.72107600

H -0.96044300 1.06483700 1.73601000

H 0.74340400 0.91897300 2.20891400

25

TS6a

C -2.25439700 2.08210100 0.79635500

C -1.06064900 1.71416300 -0.02485800

H -2.01070000 2.88227900 1.49152200

O -2.19903000 -1.09351600 1.05437300

H 2.44753300 1.06942400 -1.95192900

C -1.22968600 0.54870400 -1.00280800

H -0.80361300 0.82109500 -1.96889000

O 0.01132600 2.27214600 0.07877000

H -1.01496600 -1.49285800 -1.02287800

O 1.85422000 0.74499700 -1.27202000

H 1.40694000 1.50041000 -0.85464300

H -3.07110900 2.39054900 0.13638700

H -2.59915100 1.17862900 1.31255400

O -2.33467300 -2.24079600 -1.02974700

H -2.47139300 -1.77590900 0.13514800

C -0.86033800 -0.99503300 0.94832900

C 3.19635500 -0.62107200 0.51495800

O 4.21722900 -0.23614700 0.14282500

O 2.19786600 -1.03932600 0.92457600

H 0.48824700 -0.57696700 -0.69072200

N -0.51633800 -0.65527600 -0.51636200

H -2.27360100 0.26735000 -1.12094900

H -2.47258700 -3.18261700 -1.13313900

H -0.30258900 -1.92494800 1.12685000

H -0.43185500 -0.20155000 1.57315200

25

Pa

C 3.76679700 -0.43297800 0.77564700

C 2.78907000 0.23596300 1.70784800

H 4.76278500 -0.43471800 1.21399800

O -0.49111300 0.67490400 -1.51277400

H 1.24160300 2.55532200 4.65540700

C 1.33399700 -0.12201500 1.51820700

H 1.23022400 -1.17452900 1.83187800

O 3.15967200 1.02544300 2.54862600

H -0.50666500 0.42789800 2.07539700

O 1.56156300 1.67021700 4.85022400

H 2.31880400 1.54358300 4.26389800

H 3.45681700 -1.44754700 0.52018800

H 3.79292600 0.14712300 -0.15213100

O -1.54431700 -0.69792800 0.69386200

H -1.21894600 -0.50665700 -0.19753400

C -0.45536300 1.73038000 -0.93277800

C 1.59783800 3.14353600 1.55832700

O 1.78269300 2.78636300 0.47144400

O 1.44744900 3.60216600 2.60665900

H 0.64377400 0.84356900 3.18528100

N 0.43439800 0.79544300 2.18912200

H 1.13101500 -0.12551200 0.44099700

H -2.43455600 -1.04503400 0.61286500

H -0.72079000 1.80863500 0.13304700

H -0.14468300 2.65018300 -1.45241900

25

Reactants in Path b

C 2.38438400 -0.17294400 -1.49023200

C 1.41148500 -0.44332900 -0.38578800

H 2.12491000 -0.76467600 -2.36802500

O 2.87132400 -0.86118700 1.78857300

H 3.07848200 -0.65028500 2.70150300

C 1.05370900 0.72460200 0.53073300

H 0.33397400 0.44068700 1.32263900

O 0.95995700 -1.54267300 -0.13068100

H 2.26774900 -1.61058600 1.78279000

O 1.49495700 1.83128100 0.40842700

H 0.32731400 -2.18647900 -1.83361800

H 2.43384100 0.88845300 -1.72512600

H 3.36675600 -0.48653100 -1.12461600

O -0.00455100 -1.97689000 -2.71952800

H -0.33271100 -2.78488500 -3.11795000

C -0.20762100 1.81855300 -2.34762200

C -1.38842100 2.56210200 -2.93441000

O -0.99423700 3.55121000 -3.75270400

O -2.54003000 2.33357300 -2.69112400

H -1.78740700 4.00798100 -4.06524100

N -0.57906500 0.64161800 -1.59422300

H -1.46210200 0.80189000 -1.11914800

H -0.69426300 -0.17176700 -2.19700600

H 0.47384500 1.55074200 -3.15739600

H 0.33736300 2.52369800 -1.70614600

25

TS1b

C 2.32907100 0.05618700 -1.42571400

C 1.00788100 -0.01662100 -0.67146500

H 2.26675300 -0.56421600 -2.32269600

O 2.66840400 -1.02357300 1.47255400

H 2.61178600 -1.28679600 2.39288700

C 0.97198900 0.95242600 0.51974000

H 0.53439900 0.52099600 1.43709600

O 0.64787900 -1.26775900 -0.28339200

H 1.96826000 -1.48530700 0.98009500

O 1.32456400 2.09687500 0.44260300

H 0.35547600 -1.81648800 -1.34197200

H 2.60541600 1.07879700 -1.68683800

H 3.09662100 -0.34600300 -0.76481200

O 0.00485700 -1.83183900 -2.49721500

H -0.73751600 -2.41274500 -2.67215500

C 0.09783700 1.69543100 -2.38790600

C -1.23656700 2.24325800 -2.83312700

O -1.08366400 3.24106900 -3.70432300

O -2.29709400 1.84821000 -2.43788500

H -1.95980300 3.58099800 -3.93539100

N -0.12079800 0.46275100 -1.62756200

H -0.98848800 0.53655200 -1.09230200

H -0.24086500 -0.50463200 -2.27188700

H 0.72397000 1.49141600 -3.25617100

H 0.59929100 2.44650900 -1.77148600

25

IM1b

C 6.14295500 2.66990700 5.64059900

H 5.67443900 1.68879000 5.71985800

H 7.20335500 2.52205100 5.42844800

H 5.68717200 3.22611300 4.81961900

C 5.98453500 3.42039000 6.96018500

O 6.63898300 2.68030900 8.01503900

C 4.48320600 3.49165600 7.29550800

H 3.99893300 4.45360600 7.05971900

O 3.85734700 2.55451300 7.71886300

N 6.48403200 4.75058000 6.89634400

H 6.41344900 1.72757500 7.96001600

H 6.36580500 5.23632100 7.77828800

C 7.67493500 5.10662000 6.15529700

H 7.74373700 6.19456900 6.12956300

H 7.57407900 4.80098100 5.11093200

C 9.06032400 4.61721800 6.61082700

O 9.12021000 3.64595700 7.51983800

H 8.24276300 3.32291100 7.80413500

O 10.04618300 5.09549600 6.12856800

O 5.43647000 0.30232900 7.75310800

H 5.42243800 -0.54692300 8.19740000

H 4.58762900 0.75154800 7.86899300

O 5.43685000 4.59763800 9.51843500

H 5.90162100 3.74918100 9.49233700

H 4.74032200 4.54483700 10.17586100

25

TS2b

C 7.85678100 2.50189300 6.00391400

H 8.66334400 1.79834300 6.19432300

H 7.93345200 3.35786800 6.67847400

H 7.93330900 2.87271900 4.97794300

C 6.54160600 1.81564400 6.22422500

O 6.42669000 0.64284200 6.48143600

C 5.29643300 2.67102600 6.14933500

H 4.34670200 2.17356000 6.31593400

O 5.49886400 3.05146500 8.07457300

N 5.30602000 3.77302500 5.46190000

H 4.94181400 2.36481000 8.48161500

H 6.18879200 4.24634900 5.22902700

C 4.17390300 4.69646300 5.41137300

H 3.25508700 4.13413900 5.58066200

H 4.15947400 5.17284100 4.43315700

C 4.38804800 5.75896400 6.55112300

O 4.31761700 5.29654700 7.71144700

H 5.01752500 3.94067600 8.07871300

O 4.65252600 6.90402800 6.17468900

O 4.19852800 0.70632400 8.17672500

H 4.87698600 0.23209200 7.67754200

H 3.72004400 0.08501400 8.72829900

O 6.86698800 5.91257100 4.88511900

H 7.71252700 6.34038300 5.02856800

H 6.16720700 6.50599100 5.22721300

25

IM2b

C 6.02214300 3.25767200 4.71787300

H 5.86725600 2.18176100 4.63323100

H 6.98317200 3.51609900 4.26905800

H 5.23710700 3.78625300 4.17533600

C 6.01120600 3.67162200 6.18149700

O 7.26837600 2.41751000 9.87186200

C 4.65405500 3.33630500 6.78923500

H 3.93760200 4.17418800 6.84443600

O 4.34820400 2.21789800 7.09641700

N 6.34815200 5.07022000 6.36376900

H 6.89293200 1.59999500 9.48850900

H 5.95077800 5.33298100 7.26494600

C 7.79449000 5.04934500 6.56873500

H 8.12032900 5.82175500 7.26209100

H 8.34753400 5.16985400 5.63282100

C 8.07648400 3.66762800 7.14349400

O 7.00633300 2.88676900 6.88477500

H 8.14783400 2.51618900 9.49035600

O 9.04184100 3.28007500 7.73012300

O 6.06578100 0.44574500 8.48133500

H 5.28362300 0.01940200 8.83889300

H 5.76206200 1.00249900 7.75167100

O 5.69361100 4.37131100 8.99040400

H 6.27888300 3.65360700 9.34101000

H 5.18390400 4.70266100 9.73238100

25

TS3b

C 6.31940600 2.81329700 4.80489200

H 6.56807300 3.65421700 4.14861200

H 5.50661300 2.24443500 4.35674100

H 7.20182800 2.17538000 4.88241000

C 5.88294700 3.30617400 6.13887500

O 6.80750600 2.30802700 9.90329000

C 4.60269700 2.91019900 6.72799400

H 4.35401000 3.39349100 7.69032900

O 3.87969700 2.07407700 6.23178100

N 6.63333300 4.08392800 6.88362800

H 6.41741800 1.58638900 9.37286500

H 6.22850700 4.41001400 7.78927900

C 7.95842700 4.34816400 6.73373500

H 8.32688400 5.15602200 7.34969200

H 8.36373900 4.24246900 5.73450900

C 8.74205900 2.66702800 7.63376000

O 8.21638300 1.75911900 7.06559200

H 7.75540100 2.16963100 9.97298800

O 9.48895800 3.05455600 8.46460500

O 5.89341500 0.56496400 8.04126100

H 5.04832900 0.53346200 7.58350000

H 6.59087500 0.70693400 7.38898400

O 5.72149400 4.61609500 9.36019700

H 6.10994600 3.81149300 9.77446600

H 5.66984300 5.33110100 9.99512800

25

IM3b

C 5.67566400 3.89032100 4.30301800

H 5.32586200 2.89652900 4.02806800

H 6.58490200 4.10876300 3.72880400

H 4.91569700 4.62293200 4.00340600

C 5.92630100 3.91368200 5.76567100

O 6.78681600 2.51907200 10.09806600

C 5.76166100 2.81128500 6.61732900

H 6.01649500 3.00233200 7.67592700

O 5.37552800 1.67916300 6.28860500

N 6.38311000 5.05654400 6.35334600

H 6.31691100 1.79521200 9.62744800

H 6.52536500 5.00705400 7.38403400

C 6.64974500 6.20394200 5.80118300

H 7.01105000 7.00008300 6.43349100

H 6.51750600 6.34014400 4.73833500

C 8.50212000 0.84917800 8.49581000

O 8.36398900 1.34846500 7.46594100

H 7.40220800 2.10849700 10.70918400

O 8.69338900 0.35701800 9.52587500

O 5.84947400 0.52379100 8.59207400

H 5.20913500 -0.13131200 8.87334200

H 5.62596700 0.80648500 7.66605800

O 6.62372600 5.00213000 9.11175400

H 6.82904900 4.12321100 9.49514100

H 5.85726600 5.32920000 9.58831600

25

TS4b

C 5.49282600 3.77846200 4.27850100

H 5.16298000 2.76433100 4.04643000

H 6.24006300 4.05308400 3.52802800

H 4.63579100 4.44900600 4.14396600

C 6.04189300 3.84689500 5.68383600

O 7.42199500 6.10285400 8.60818800

C 5.65674800 2.85237200 6.63277700

H 5.15978000 1.96820100 6.18920400

O 5.92016600 2.82057300 7.84206300

N 6.40568700 5.09964400 6.22668800

H 8.44201500 4.60227400 8.61358600

H 6.87465300 5.71936900 7.88976300

C 6.49729500 6.14264500 5.50449000

H 6.81112900 7.06963800 5.97898500

H 6.28618700 6.16428900 4.43407200

C 7.68116800 0.86841000 8.63427900

O 8.04367300 0.76305600 7.53597100

H 6.84762400 6.31007400 9.34791800

O 7.34776400 0.92876800 9.73185500

O 8.39688600 3.65861300 8.36464500

H 8.64009600 3.39642900 6.97773500

H 7.43535700 3.45592500 8.40465000

O 8.56527000 3.08214900 5.96928100

H 7.46374800 3.29712200 5.72142700

H 8.70636900 2.12639600 5.96873200

25

IM4b

C 1.32729600 -2.61387300 0.28084200

C 0.73140200 -1.22266100 0.03919100

H 0.88700900 -3.05460600 1.17602700

O 2.39500400 2.34791000 0.05520600

H -0.77333000 1.36948200 -0.13549900

C 1.34883400 -0.66144000 -1.23866200

H 0.72418400 -0.78289300 -2.14344200

O -0.13255200 1.49597400 -0.85960100

H 1.48651800 2.21812100 -0.25839900

O 2.45829300 -0.20888100 -1.31148200

H -0.62748100 1.69882300 -1.65600100

H 1.13317600 -3.27852700 -0.56387700

H 2.40783900 -2.54143300 0.41771400

O -1.58962300 0.71276600 1.30173000

H 2.92279000 1.77817700 -0.51177600

C 2.03544600 0.04779900 1.54450700

C -3.35588100 -0.16291800 -0.38499700

O -2.53568900 -0.28420000 -1.19344200

O -4.21784700 -0.05655300 0.37278100

H -0.72585600 0.29881200 1.54696500

N 0.88526600 -0.35502700 1.20537300

H -0.34209200 -1.31605100 -0.14248300

H -1.90869400 1.23152700 2.04256500

H 2.95407000 -0.24386800 1.03863400

H 2.12174500 0.73006300 2.38514400

25

TS5b

C -3.18650700 0.44141900 -0.62971400

C -1.79036200 -0.20025400 -0.54018900

H -3.28071300 0.94604800 -1.59091000

O 0.43602600 1.63042300 1.30741400

H 2.27189600 0.92010900 -0.74175800

C -1.71216500 -0.95388000 0.77179200

H -2.43162400 -1.78882900 0.87775400

O 2.59703700 1.46899800 0.07314700

H 1.39687500 1.56377200 0.90634500

O -0.96218200 -0.70324400 1.68350000

H 3.28350400 0.95518700 0.50508800

H -3.97572100 -0.30947700 -0.55338100

H -3.34769100 1.17462400 0.16303700

O 1.50564500 0.07813800 -1.68150700

H 0.16458400 0.76007400 1.65259400

C -0.54393700 1.83367200 -0.08341100

C 1.21941800 -1.79717900 0.04266400

O 0.45132100 -2.38786300 -0.58834800

O 1.96474700 -1.27243700 0.75316700

H 0.32282400 0.35326800 -1.36174600

N -0.70189000 0.71262100 -0.79185200

H -1.73614400 -0.97734000 -1.31221000

H 1.68193100 0.11917800 -2.62236400

H -1.40487700 2.26936200 0.41949500

H 0.10894200 2.57087900 -0.54482200

25

IM5b

C 0.00146500 2.00578100 -0.79824700

C -0.89446400 0.91950300 -0.21811700

H 0.94719900 1.54951700 -1.09141200

O -0.59119900 -1.90677100 0.50697800

H -3.67686500 -2.20533700 -0.80144600

C -2.24115600 1.49874200 0.14964200

H -2.20142600 2.40482800 0.78312600

O -2.90505400 -1.63958900 -0.86139800

H 0.80404700 -1.39430100 -0.48834000

O -3.30790300 1.02395100 -0.16071200

H -3.19418300 -0.71615300 -0.77330700

H -0.45066900 2.46651200 -1.67854300

H 0.19406500 2.77954600 -0.05097100

O 1.49167900 -0.91233100 -0.98524700

H -1.38753400 -1.98300600 -0.05249100

C -0.81805800 -0.86445800 1.44623600

C 3.53700800 0.12224100 0.12772200

O 2.89011100 0.61796600 0.95007400

O 4.24948100 -0.33361300 -0.65612200

H 0.67962100 0.45179600 1.00058800

N -0.33510900 0.43051000 1.03655700

H -1.03096200 0.12532900 -0.96287700

H 1.73575200 -1.44402700 -1.74539800

H -1.89295100 -0.79782900 1.64520100

H -0.30601800 -1.13820100 2.36797300

25

TS6b

C -0.95224900 2.24390700 -0.28718500

C -1.02488300 0.96771000 0.55153500

H -1.71343700 2.17463400 -1.06498100

O -2.90212900 -1.16196900 0.34196800

H 1.88467100 -2.65049700 0.21195400

C -0.07697500 1.00383700 1.71890100

H -0.16627700 1.90280200 2.35685100

O 1.60173700 -1.73517600 0.17166800

H -1.38767700 -0.07172700 -1.18455200

O 0.73153300 0.15263000 1.98942000

H 1.43059300 -1.41501600 1.06728600

H -1.16035500 3.11898700 0.33141000

H 0.03106000 2.36154400 -0.74368000

O -2.83459500 0.04639300 -1.71355900

H -3.10236500 -0.63818100 -0.70546700

C -1.59691800 -1.46274100 0.29751400

C 3.02324200 0.24349500 -0.77689200

O 4.01131400 -0.34544500 -0.82503500

O 2.05722400 0.88312900 -0.74803900

H 0.15539400 -0.46917200 -0.42874600

N -0.82810600 -0.24037800 -0.27051300

H -2.04138600 0.87358400 0.95618000

H -3.20953000 -0.26199200 -2.53907500

H -1.16575000 -1.67138600 1.28383300

H -1.31217800 -2.26468600 -0.40204100

25

Pb

C 1.99844200 3.57864800 -0.21507000

C 0.77919000 2.72886000 -0.51713600

H 2.32857000 4.11262000 -1.10639200

O -2.26509600 3.62450300 0.17266700

H 3.71435700 2.93785700 -2.22387100

C 1.06430700 1.64613400 -1.53624600

H 0.18260700 1.04420900 -1.83661900

O 3.89744500 3.35231000 -3.07227600

H 3.21793400 0.64772700 -3.57072200

O 2.15575700 1.37282500 -1.97257800

H 4.19603300 2.61812900 -3.63176100

H 1.75628700 4.30666800 0.55921500

H 2.82377300 2.95915200 0.14689500

O 3.83418400 0.85192700 -4.28448400

H -0.58748500 1.57995500 0.52781900

C -1.66624400 3.98089800 1.14881600

C 1.57165000 3.50010400 -4.21680300

O 1.05764100 4.00609800 -3.31389600

O 1.98677900 2.97959700 -5.16176600

H 0.95132300 1.46803300 1.10297100

N 0.26383300 2.10375600 0.70906200

H -0.02919800 3.34506200 -0.92624500

H 3.30007800 1.09583300 -5.04560600

H -0.82970100 4.69963200 1.09328000

H -1.92587200 3.61832600 2.15871000

25

Reactants in Path a[w]

C 2.01796400 -0.44394900 -1.75739400

C 1.21328700 -0.61996900 -0.50783800

H 1.72192800 -1.18231300 -2.50102800

O 3.19586500 -0.82892300 1.25191300

H 3.62115000 -0.23105500 1.87375300

C 1.14999100 0.56941900 0.44822200

H 0.62722400 0.33668600 1.39157700

O 0.66332000 -1.64582900 -0.17724000

H 2.83309400 -1.55133800 1.77351700

O 1.59524200 1.66050000 0.20205400

H -0.11947200 2.76886500 0.74584800

H 1.91080800 0.56574700 -2.14975900

H 3.06872300 -0.59809600 -1.49666500

O -1.08030500 2.79982700 0.82237000

H -1.34850500 1.91852600 1.11792000

C -1.86835500 -0.44984200 -1.23640000

C -2.08858700 -0.72272500 0.22862900

O -2.64393400 -1.91266100 0.43669700

O -1.84306600 0.04913200 1.12575300

H -2.80939600 -2.02160900 1.38490700

N -0.94043400 0.65151900 -1.40770000

H -0.81753100 0.85113600 -2.39386000

H -1.28083200 1.50039900 -0.96307800

H -1.45719300 -1.35519600 -1.68621300

H -2.86138200 -0.29071600 -1.67842200

25

TS1a[w]

C 2.48554200 -0.27286800 -1.92665600

C 1.45139500 -0.87144300 -1.01883300

H 2.70337400 -0.95984700 -2.74186000

O 3.53577300 -0.35309900 1.13229300

H 2.92552500 0.38256900 0.93710000

C 0.77842300 0.06891900 -0.00540500

H 0.54093100 -0.52699700 0.88796700

O 1.11356900 -2.03168400 -1.06162400

H 3.24623400 -0.73159700 1.96651500

O 1.49314300 1.19791400 0.24360700

H 0.64373600 2.02435700 0.67396500

H 2.16401600 0.69713600 -2.30823700

H 3.38811000 -0.10057300 -1.33609800

O -0.41933100 2.50434300 0.78143100

H -0.76134000 2.36091000 1.67013000

C -1.62967300 -0.56105200 -0.45308600

C -2.14946400 -0.60512400 0.96610300

O -3.04992100 -1.57180200 1.10676100

O -1.80477700 0.13545800 1.84805900

H -3.37814100 -1.57417500 2.01837800

N -0.58658600 0.46252000 -0.57316400

H -0.48115000 0.74573400 -1.54673100

H -0.78736400 1.50023600 0.07060500

H -1.25395900 -1.54627600 -0.73537800

H -2.46030500 -0.31482100 -1.11531800

25

IM1a[w]

C 7.73698300 3.33359300 6.56307600

H 8.46080900 2.63262400 6.97335900

H 7.65077600 4.20231400 7.22124400

H 8.06236500 3.71692200 5.59482600

C 6.40035100 2.65819300 6.44161300

O 6.18701200 1.56361000 6.90665800

C 5.30028800 3.37959300 5.63960200

H 5.41275900 3.03067300 4.60050200

O 4.01384900 3.01310900 6.11128100

N 5.49092300 4.78903100 5.66296800

H 3.82655200 2.06819200 5.92849100

H 5.52175300 5.17252600 6.60685500

C 4.59609300 5.58130600 4.82866700

H 4.23736800 4.96154500 4.00070700

H 5.12390800 6.43534600 4.40416200

C 3.38544800 6.15709500 5.57062400

O 2.68758700 5.30240700 6.32509400

H 3.04615400 4.38843600 6.26440400

O 3.09278200 7.31868900 5.51516500

O 4.01733100 0.37450600 5.72639200

H 4.78994100 0.34710300 6.30765800

H 3.38206200 -0.28356300 6.01412100

O 4.75739500 6.46295200 7.99514500

H 3.86054800 6.12032300 8.08703000

H 4.65243200 7.35466500 7.64814600

25

TS2a^{w}

C 7.85678100 2.50189300 6.00391400

H 8.66334400 1.79834300 6.19432300

H 7.93345200 3.35786800 6.67847400

H 7.93330900 2.87271900 4.97794300

C 6.54160600 1.81564400 6.22422500

O 6.42669000 0.64284200 6.48143600

C 5.29643300 2.67102600 6.14933500

H 4.34670200 2.17356000 6.31593400

O 5.49886400 3.05146500 8.07457300

N 5.30602000 3.77302500 5.46190000

H 4.94181400 2.36481000 8.48161500

H 6.18879200 4.24634900 5.22902700

C 4.17390300 4.69646300 5.41137300

H 3.25508700 4.13413900 5.58066200

H 4.15947400 5.17284100 4.43315700

C 4.38804800 5.75896400 6.55112300

O 4.31761700 5.29654700 7.71144700

H 5.01752500 3.94067600 8.07871300

O 4.65252600 6.90402800 6.17468900

O 4.19852800 0.70632400 8.17672500

H 4.87698600 0.23209200 7.67754200

H 3.72004400 0.08501400 8.72829900

O 6.86698800 5.91257100 4.88511900

H 7.71252700 6.34038300 5.02856800

H 6.16720700 6.50599100 5.22721300

25

IM2a[w]

C 7.35246400 2.95518000 5.68021400

H 8.27306600 2.51031100 5.30898900

H 6.87286500 2.28090000 6.39410900

H 7.55846200 3.88659300 6.21420800

C 6.41913600 3.19342000 4.52416600

O 6.63513300 2.84276600 3.39665800

C 5.12740500 3.95901700 4.81995800

H 4.36844500 3.67605200 4.08711500

O 2.96062100 7.26381800 8.33150300

N 5.32389700 5.38986900 4.83191400

H 2.26734200 7.49877500 8.95032000

H 6.20222300 5.57817100 5.31229700

C 4.27123600 5.84682000 5.73488600

H 3.33347600 6.01640200 5.19517400

H 4.54197400 6.75317500 6.27081400

C 4.08335600 4.67988200 6.69258300

O 4.62135500 3.59137500 6.13399400

H 3.00115400 6.29269800 8.26014600

O 3.52718600 4.65488000 7.76005000

O 5.55551500 7.89623200 8.27737700

H 4.59057300 7.79165000 8.40466300

H 5.72477800 8.77144100 7.92515100

O 6.58671100 5.53771600 7.47121000

H 6.54611400 4.92373900 8.20855100

H 6.36072000 6.42319400 7.81783600

25

TS3a^{w}

C 3.77003900 -0.53153300 0.28742100

H 4.39830000 0.18697500 0.80826100

H 4.24660000 -0.82832600 -0.65033700

H 3.65940400 -1.43386300 0.89473100

C 2.42204400 0.07727200 0.01644800

O 2.13855100 1.21778400 0.33722500

C 1.43167700 -0.78010700 -0.65406300

H 1.58736200 -1.84169900 -0.79829000

O -0.35397900 0.77316300 1.80522900

N 0.32801100 -0.23374100 -1.07270200

H -0.21959000 -0.18135200 1.84278600

H 0.19515100 0.81108600 -0.95928000

C -0.79836300 -0.87613400 -1.48661300

H -0.64608500 -1.85258800 -1.93663600

H -1.51833900 -0.22352400 -1.96190300

C -1.60616100 -1.50043500 0.16174200

O -0.71695400 -1.91885000 0.84733000

H 0.44816600 1.17117200 1.44548000

O -2.78350000 -1.33426800 0.05268000

O -2.58482100 1.56101300 0.45016300

H -3.09279400 0.77436100 0.22431200

H -1.95930400 1.29674500 1.14904200

O -0.58911300 2.23229400 -1.12148100

H -0.15081200 3.03619100 -0.83644400

H -1.44102600 2.13755300 -0.63159800

25

IM3a^{w}

C 8.92595100 4.27296700 7.83439700

H 9.62905200 3.50842600 8.16958200

H 8.75854400 4.94939100 8.67616600

H 9.36665900 4.83056700 7.00774700

C 7.62021300 3.60335000 7.47388500

O 7.05835400 2.88177200 8.32615100

C 7.08527700 3.85439600 6.19820100

H 7.57228900 4.44189200 5.43412800

O 4.03376700 1.65527400 7.04106800

N 5.92302400 3.20194800 5.86044800

H 3.87362800 2.21690100 7.83148800

H 5.51690100 2.57751400 6.58516500

C 5.23150800 3.23105700 4.77393800

H 5.55242500 3.84690500 3.94389600

H 4.33340800 2.63181500 4.74544900

C 6.80126600 3.67261000 10.89723000

O 6.71976900 2.59192800 11.27986700

H 4.14200200 0.75363200 7.35173300

O 6.89411300 4.78241300 10.56916700

O 4.44491700 3.29025500 9.08144500

H 4.45048000 4.22542900 8.81687000

H 5.36180200 2.99971700 8.93725600

O 5.38354700 5.73907100 8.23377000

H 5.94529800 5.44636900 7.50437200

H 5.96177700 5.85324200 8.99549000

25

TS4a^{w}

C 0.74728700 2.64588700 -0.72165200

H 0.91811300 2.89270100 -1.77144700

H 1.72619500 2.49132400 -0.26250100

H 0.24650000 3.48359800 -0.23680800

C -0.04272900 1.35533200 -0.66144200

O 0.48298400 0.33408500 -1.14348700

C -1.27645800 1.37587000 0.05375400

H -1.70786800 2.34674700 0.29919400

O -2.03544300 -2.39233600 -0.78347900

N -2.17522300 0.30579300 -0.12549900

H -0.52872900 -2.34795700 0.20584200

H -2.05750400 -1.41166000 -0.77241100

C -3.38294300 0.40789800 0.25182100

H -3.78924900 1.32042300 0.69909200

H -4.04610000 -0.44510300 0.13060400

C 2.77995600 -0.51462000 0.06044400

O 3.10010900 -1.15023500 -0.84088100

H -1.99984700 -2.68325700 -1.69686700

O 2.50908800 0.12256300 0.99359200

O 0.22562000 -1.76881100 0.43306500

H -0.02673000 -0.84713000 1.45852100

H 0.28994400 -1.15085400 -0.33491000

O -0.14500900 0.03997200 2.04917100

H -0.59079300 0.77069600 1.31141300

H 0.74316300 0.33581300 2.28737100

25

IM4a[w]

C 3.75992300 -0.71028500 -0.44412300

C 2.39618400 -0.13937300 -0.18374100

H 4.33573300 -0.03409200 -1.07209000

O 0.10184800 2.85774800 0.31779700

H -2.02765700 1.28209800 -0.99749800

C 1.59074500 -0.79875200 0.92621800

H 2.09621600 -0.59273400 1.87992300

O 1.94406600 0.78783200 -0.81895800

H -0.83102200 2.63105900 0.16687800

O -2.42721200 1.94201100 -0.39702100

H -3.03167900 1.46044800 0.17403700

H 4.28847100 -0.90433800 0.49078000

H 3.64618100 -1.66952500 -0.95694800

O -0.89966100 0.11094400 -1.66082800

H 0.61573500 2.14984100 -0.08976400

C -0.19873900 0.37250400 1.85359100

C -2.07360600 -1.79944800 -0.02331500

O -2.74624000 -0.93096000 0.33894600

O -1.42824900 -2.68828600 -0.37496300

H -0.39135600 -0.06038100 -0.84312700

N 0.22202500 -0.33109600 0.89339100

H 1.61033900 -1.88189200 0.77332400

H -0.26774300 0.37944400 -2.33365200

H -1.21353100 0.75899700 1.81641800

H 0.41267800 0.61656500 2.72641700

25

TS5a^{w}

C -3.35543300 0.16655300 -0.27315300

C -1.94489400 -0.34327300 -0.30266400

H -3.74161200 0.27297600 -1.28443100

O 1.10453600 -1.72670200 -1.22219400

H 2.51477900 0.29164800 0.32813900

C -1.42338900 -0.92340900 1.00467000

H -1.92074500 -1.89276300 1.13729700

O -1.26176900 -0.29056200 -1.30453300

H 1.87893500 -1.07758900 -1.06799600

O 2.94588600 -0.10794200 -0.55032200

H 3.04067100 0.62197600 -1.16851000

H -4.00027200 -0.48566600 0.31810500

H -3.34701700 1.14637300 0.21368100

O 1.72666300 0.74273200 1.42693600

H 0.33615900 -1.19896200 -1.50538000

C 0.63583700 -2.08427300 0.53292900

C 0.14692500 2.02637400 -0.14667100

O -0.70258800 2.08531200 0.63692500

O 0.93450900 2.00594800 -0.99060100

H 0.68413000 -0.21400400 1.36813400

N 0.00942800 -1.06801100 1.08349800

H -1.76615500 -0.28749500 1.82388200

H 2.17292400 0.77897600 2.27603200

H 1.67235400 -2.21783400 0.82123100

H 0.07769500 -2.98368300 0.29498800

25

IM5a[w]

C -3.76711900 -1.08224600 0.49240400

C -2.56515500 -0.23711700 0.17206500

H -4.61042100 -0.45274900 0.76842800

O -0.32828000 2.13414500 -0.68719500

H 1.67167400 0.66268100 1.64808100

C -1.45211200 -0.93108500 -0.60339700

H -1.84076300 -1.10026200 -1.61565600

O -2.49348900 0.92460600 0.51609000

H 1.11692900 2.32587000 0.31924400

O 1.94362700 2.18936400 0.82417400

H 2.63561500 2.00672100 0.18108800

H -4.03104700 -1.72666100 -0.34808800

H -3.51835600 -1.73541800 1.33381900

O 1.42456000 -0.26487100 1.83918600

H -1.14952000 1.97107400 -0.19362200

C -0.05127400 0.93932100 -1.42452400

C 2.41259600 -1.42565800 -0.34000700

O 2.84312900 -0.38343100 -0.60004900

O 2.03152900 -2.48971400 -0.10858700

H 0.28650000 -0.21915500 0.23505200

N -0.16787200 -0.27688500 -0.67641700

H -1.32031700 -1.93076700 -0.17642500

H 0.83877600 -0.26038800 2.60075700

H 0.97660900 1.05976500 -1.77059600

H -0.71079900 0.87740700 -2.29537200

25

TS6a^{w}

C 3.88922100 -0.34620400 0.67527900

C 2.63825000 0.23986100 1.25921400

H 4.75637200 0.22373900 1.00089500

O -0.77019900 0.50377500 -0.55195700

H -0.00441000 2.10247500 4.33058200

C 1.37642800 -0.58812300 1.10114800

H 1.48597900 -1.51296000 1.67216600

O 2.60901500 1.30860200 1.82574300

H -0.64616900 -0.53214000 1.58514200

O 0.67137500 1.52745300 3.96052400

H 1.41557200 2.08686600 3.71143100

H 3.99629200 -1.39515800 0.95768900

H 3.81954900 -0.31075900 -0.41546400

O -1.89343300 -1.17063600 0.75322500

H -1.44125600 -0.30147600 -0.08518600

C -0.30232000 1.16579300 0.53897600

C 2.20833900 3.94192100 1.72639400

O 2.33279000 3.96041700 0.58072900

O 2.07561100 3.94685200 2.87551900

H 0.34292600 0.58654900 2.48226400

N 0.18756200 0.14087600 1.56077500

H 1.22294800 -0.86164100 0.05516600

H -2.75454800 -0.90686700 1.08894700

H -1.06534900 1.73287000 1.09051200

H 0.53795200 1.82243700 0.29987300

25

Pa[w]

C 3.90681100 -0.40529600 0.82309100

C 2.87213100 0.20582200 1.72568800

H 4.88758200 -0.37620800 1.29351500

O -0.45776700 0.65623400 -1.33277500

H 1.10581000 2.51985900 4.53377300

C 1.43868800 -0.19151300 1.47050000

H 1.36516200 -1.25421900 1.75527500

O 3.17708000 0.98618000 2.60621800

H -0.44442500 0.31592800 1.94746600

O 1.56231500 1.72890000 4.83531000

H 2.22588300 1.55314700 4.15209000

H 3.64439300 -1.42660900 0.54445300

H 3.93871000 0.18766800 -0.09638800

O -1.70970900 -0.73682700 0.77278900

H -1.32443900 -0.47278900 -0.07632700

C -0.55438300 1.76834600 -0.87526700

C 1.56900800 3.06856100 1.48543200

O 1.80754700 2.68218700 0.42091900

O 1.36605200 3.54341300 2.51891800

H 0.63301600 0.66122100 3.14093700

N 0.48766800 0.68058600 2.13530700

H 1.27223600 -0.16469600 0.38838900

H -2.63107400 -0.46389800 0.75481500

H -0.99057300 1.94065800 0.12053500

H -0.21273000 2.64715700 -1.43937800

25

Reactants in Path b[w]

C 2.44975900 -0.23957500 -1.48868900

C 1.45342300 -0.49066100 -0.39958400

H 2.20285800 -0.83818300 -2.36541100

O 2.78944900 -0.84672300 1.93925200

H 2.79115000 -0.46580500 2.82202100

C 1.09831500 0.68376400 0.50865300

H 0.32729300 0.43115900 1.25856900

O 0.97573900 -1.58110400 -0.15677200

H 2.15133500 -1.56741200 1.95218300

O 1.60128700 1.77154700 0.42292600

H 0.34580100 -2.22744300 -1.90520400

H 2.50878400 0.81681600 -1.74248300

H 3.42814800 -0.55620500 -1.11518000

O 0.03091600 -2.02946000 -2.79911200

H -0.68086400 -2.64641500 -2.98998200

C -0.15665100 1.86592800 -2.30658300

C -1.33749300 2.51397800 -2.99902600

O -0.96350600 3.59452700 -3.69358700

O -2.47531000 2.13269800 -2.93055900

H -1.74630200 3.99056500 -4.10356400

N -0.47641500 0.64515600 -1.60383800

H -1.36269500 0.73626400 -1.11725000

H -0.54656100 -0.15352900 -2.23346300

H 0.61482300 1.67655400 -3.05587800

H 0.26480300 2.60287400 -1.61247400

25

TS1b[w]

C 2.36142600 0.08588500 -1.47101500

C 1.05811400 0.00924100 -0.69150700

H 2.28418000 -0.52516600 -2.37251400

O 2.67868000 -1.09653100 1.58432700

H 2.32789200 -1.09272700 2.47879700

C 1.05382300 0.95772100 0.51438600

H 0.44950300 0.59613100 1.36618200

O 0.72911300 -1.25637300 -0.28671800

H 1.97700000 -1.44776700 1.00798600

O 1.60610600 2.02351400 0.51953100

H 0.37238600 -1.78915200 -1.29343200

H 2.63428000 1.10722400 -1.73653500

H 3.14324900 -0.32332900 -0.83101000

O -0.04437500 -1.84690700 -2.45887100

H -0.87782400 -2.32205600 -2.51602300

C 0.09434500 1.71162500 -2.36013500

C -1.25118400 2.19978600 -2.84521600

O -1.11458800 3.23211400 -3.66791200

O -2.30056500 1.72046500 -2.51179400

H -1.98986000 3.54111800 -3.94600000

N -0.09359000 0.47090300 -1.60009000

H -0.94957200 0.53335900 -1.04330000

H -0.23154400 -0.49435100 -2.24300700

H 0.74724700 1.53052900 -3.21286500

H 0.54918100 2.48700100 -1.73848800

25

IM1b^{w}

C 6.07335200 2.70332900 5.58074800

H 5.57953500 1.73437900 5.65705700

H 7.12256200 2.52860000 5.33704300

H 5.60978500 3.28286200 4.78114600

C 5.96459600 3.43223300 6.91733400

O 6.63617600 2.67080300 7.94054300

C 4.47453500 3.51575900 7.28622100

H 3.97651000 4.46173900 7.01624500

O 3.87102700 2.59724500 7.77613400

N 6.48035000 4.76123900 6.86779700

H 6.36905100 1.72210000 7.89966500

H 6.38265700 5.22195500 7.76657500

C 7.69530000 5.09057900 6.15715800

H 7.77056800 6.17662100 6.09440000

H 7.63407000 4.74204200 5.12326700

C 9.05158400 4.61711400 6.69371500

O 9.07970500 3.63116400 7.58158500

H 8.19068300 3.25949600 7.79208400

O 10.07172900 5.12243200 6.29804100

O 5.46993400 0.28702500 7.74918800

H 5.61371200 -0.43368900 8.36893900

H 4.61159100 0.68584600 7.94422600

O 5.53784000 4.59553300 9.58303900

H 5.93522200 3.71992100 9.47200200

H 4.71969800 4.47784700 10.07362700

25

TS2b^{w}

C 6.71389700 2.56017900 5.91201800

H 6.32332800 1.55853100 6.07704800

H 7.78800800 2.56117700 6.09043600

H 6.52129000 2.85403700 4.87685100

C 6.02831000 3.53226800 6.82395200

O 6.53963700 2.79417000 8.50723200

C 4.52732400 3.31601600 7.03072200

H 3.89744000 4.20119700 6.87217200

O 4.06855000 2.23265300 7.26225700

N 6.42576100 4.78852500 6.86411700

H 6.50183900 1.81033300 8.50333200

H 5.87926600 5.39578100 7.47079500

C 7.74485300 5.25525000 6.45471900

H 7.71502200 6.34220200 6.43160500

H 7.94235000 4.91997200 5.43424900

C 8.94793400 4.81723700 7.33351000

O 8.78023100 3.87350500 8.16069200

H 7.49518700 3.15924500 8.46133800

O 9.99271000 5.42422300 7.10469200

O 5.91632900 0.25120300 8.11934100

H 5.86988100 -0.44974200 8.77530600

H 5.01422400 0.49637500 7.87666000

O 4.89032900 4.83543300 9.17238700

H 5.40710600 4.05077100 9.41049200

H 4.09166600 4.84165700 9.70628500

25

IM2b^{w}

C 6.01833900 3.20823300 4.76185400

H 5.86295400 2.12951800 4.72892300

H 6.97628000 3.44678000 4.29648700

H 5.22632600 3.70621300 4.20133500

C 6.01359400 3.69015700 6.20314700

O 7.23541400 2.32429500 9.88507800

C 4.65830400 3.39501400 6.83372000

H 3.94757900 4.23679700 6.85383100

O 4.34415400 2.29204300 7.19523500

N 6.36571400 5.09231100 6.32725100

H 6.85142700 1.55973800 9.40804100

H 5.95917700 5.40744800 7.20651400

C 7.81144800 5.07010300 6.54894800

H 8.13715100 5.87612400 7.20232200

H 8.37448700 5.13189800 5.61370700

C 8.06722200 3.71702100 7.18890500

O 7.00944600 2.92873700 6.94149400

H 8.08098100 2.50180400 9.45475700

O 9.01878600 3.34186500 7.81745000

O 6.11405000 0.40988500 8.33656700

H 5.46683900 -0.17170900 8.74496100

H 5.62432200 1.00191600 7.74566200

O 5.74721900 4.41971100 8.99242800

H 6.24015400 3.66284100 9.37853100

H 5.17232500 4.77059800 9.67710900

25

TS3b^{w}

C 0.45252200 -2.23759100 0.53403500

H 0.14199100 -2.80633100 1.41667700

H 1.37198200 -2.67518100 0.15009800

H -0.33928600 -2.34634800 -0.21395500

C 0.68197000 -0.81128000 0.87863600

O 0.54200600 2.00478100 -1.35568400

C 1.98698400 -0.18111600 0.73892100

H 2.07777900 0.84054900 1.14142100

O 2.93277600 -0.73073600 0.20219800

N -0.29143000 -0.05853700 1.35155500

H 1.03428800 1.22030700 -1.66365200

H -0.05909200 0.94826900 1.50662700

C -1.59690800 -0.39047000 1.42982500

H -2.22046100 0.31738300 1.95608700

H -1.83293400 -1.44367500 1.51676700

C -2.31277000 -0.21680600 -0.54702800

O -1.34324700 -0.19934800 -1.23827300

H -0.38060900 1.77956900 -1.51679200

O -3.48327900 -0.20416800 -0.40518000

O 1.33767400 -0.47557300 -2.23059200

H 1.99732300 -0.93691500 -1.69856900

H 0.47658700 -0.85979200 -2.02455400

O 0.50717900 2.54965400 1.27102600

H 0.58834500 2.53407700 0.29250400

H -0.03156500 3.30455100 1.52216000

25

IM3b[w]

C 5.76203100 3.90351900 4.28368000

H 5.49111800 2.89266600 3.98269400

H 6.68719500 4.18150000 3.76456100

H 4.97869900 4.58802700 3.93688800

C 5.91974500 3.93547500 5.76170300

O 7.17553800 2.52184400 9.86766700

C 5.75012500 2.84157300 6.60623900

H 5.90301100 3.05231700 7.67914800

O 5.44969400 1.67423000 6.26494500

N 6.27350800 5.11741600 6.37203400

H 6.58628200 1.82243900 9.50666100

H 6.38303600 5.07700100 7.41014700

C 6.49544100 6.26700000 5.82054900

H 6.77205700 7.09358600 6.45745900

H 6.40465300 6.38740000 4.75072600

C 8.54028200 0.48514000 8.55244800

O 8.45348800 1.02404800 7.53473500

H 7.33954500 2.32010300 10.79246500

O 8.67035200 -0.07314400 9.55531000

O 5.83815600 0.55334600 8.62968500

H 5.01329900 0.23937800 9.00887100

H 5.65645500 0.83421900 7.69753400

O 6.57269000 5.02864600 9.11533700

H 6.84960500 4.14205300 9.43658400

H 5.76293100 5.25394100 9.58215800

25

TS4b^{w}

C 2.22729400 -2.21999500 -0.15030000

H 1.62980600 -3.12235000 -0.28712800

H 2.68026000 -2.27840000 0.84355400

H 3.04357700 -2.24047000 -0.88141400

C 1.36279500 -0.99081900 -0.30828600

O 1.09770300 2.94482900 -0.18162700

C 0.14496100 -1.08731600 -1.03602100

H -0.19191000 -2.12350900 -1.21828600

O -0.61185300 -0.16145000 -1.38218200

N 1.95712400 0.29364300 -0.25745700

H -0.37873200 2.28899200 0.61393100

H 1.36030200 2.02735500 -0.41291000

C 3.17153300 0.44623500 0.09173400

H 3.57121200 1.45657300 0.14165300

H 3.84195100 -0.37518400 0.34618600

C -3.02479600 -0.40314700 -0.03638100

O -2.50458400 -1.10076700 0.72681600

H 0.95798600 3.42602100 -1.00146100

O -3.57385700 0.28476700 -0.78004800

O -0.97732900 1.51748300 0.67260200

H -0.41427300 0.45866600 1.54494000

H -0.88058700 1.08490300 -0.20495200

O 0.04978600 -0.39666000 1.91320900

H 0.57601500 -0.82013600 0.97770600

H -0.62513900 -1.01332600 2.22575200

25

IM4b^{w}

C 5.48157700 4.07063200 4.17552500

H 5.61351000 3.10544600 3.68450000

H 5.74598200 4.84241800 3.45252800

H 4.42627500 4.17998900 4.43561000

C 6.37313800 4.14025800 5.41425000

O 7.26930000 5.04464400 8.87770500

C 6.13505600 2.92226000 6.28793100

H 6.14121600 1.96318300 5.74506800

O 5.93203800 2.95947500 7.47486700

N 6.29866300 5.33841700 6.23963000

H 10.04579500 3.42548900 8.66209900

H 6.93613800 5.21675300 7.97027500

C 5.65240600 6.35018800 5.85018400

H 5.62368000 7.22701400 6.49349900

H 5.11582000 6.40856400 4.90200700

C 7.44332700 1.44685200 9.09399600

O 7.72714500 0.70602900 8.25375200

H 6.67164100 4.37741500 9.23266300

O 7.14307200 2.13939700 9.96842000

O 9.19633700 3.30226700 8.23257100

H 9.00152400 2.58702300 6.66964900

H 8.59431100 4.02488700 8.51605000

O 8.77052900 2.11241300 5.84736100

H 7.42015000 4.02936800 5.10496900

H 8.53226500 1.22566000 6.13179000

25

TS5b^{w}

C -3.21968600 -0.33506800 0.57431100

C -1.81652700 0.28678500 0.53361700

H -3.35430500 -0.85431000 1.52289800

O 0.33177100 -1.76377300 -1.33282000

H 2.20282300 -0.94836200 0.71801200

C -1.66387700 1.01858700 -0.78339100

H -2.34258200 1.87800800 -0.91999600

O 2.53425000 -1.54428900 -0.09106700

H 1.27549700 -1.68149600 -0.94594000

O -0.89470100 0.71331600 -1.66329900

H 3.18607900 -1.02527900 -0.57025900

H -3.98067700 0.44195000 0.49370400

H -3.37706000 -1.04285800 -0.24080900

O 1.55971000 -0.10803700 1.67043600

H 0.07112800 -0.89402500 -1.68156700

C -0.64973000 -1.83397900 0.23591300

C 1.30975800 1.78993500 -0.05304400

O 0.54663800 2.37965600 0.58567700

O 2.05268600 1.26122400 -0.76168000

H 0.20157000 -0.32522900 1.31440800

N -0.74494000 -0.65256600 0.80640700

H -1.76638200 1.05685400 1.30979100

H 1.75369700 -0.29705300 2.59134200

H -1.52004800 -2.28598100 -0.22463600

H 0.06237900 -2.51659400 0.68579400

25

IM5b^{w}

C 1.48065300 5.44654100 -0.53623200

C 0.42627100 4.82449000 0.37050000

H 1.02679900 5.66542300 -1.50364900

O -2.31202000 3.82397400 0.05914700

H -2.85895200 5.17862600 2.96508400

C 0.99650800 4.61406000 1.75469600

H 1.96320300 4.07986400 1.78906200

O -2.27416000 5.41284600 2.23912100

H -1.86776600 4.78368100 -1.41859100

O 0.45393300 4.95536700 2.77961600

H -1.36469600 5.39527500 2.57876900

H 1.86160500 6.38007200 -0.11961500

H 2.31533900 4.75677800 -0.68032600

O -1.46205700 5.26116300 -2.16483200

H -2.44519800 4.44722200 0.80237500

C -1.19634500 3.00184300 0.38143700

C 0.29170800 4.45319500 -3.85576000

O 0.31864500 3.39962300 -3.37831900

O 0.31796900 5.48485300 -4.37114600

H 0.07590100 3.48649900 -1.12795000

N 0.06881800 3.49257000 -0.11208100

H -0.43749700 5.49736500 0.44116200

H -1.59091800 6.19921700 -2.00205100

H -1.13825800 2.89287500 1.46894100

H -1.37352900 2.02270800 -0.06145700

25

TS6b^w

C -0.87536000 2.06654500 -1.00465900

C -1.01562300 1.17528700 0.22803200

H -1.60784900 1.75210000 -1.74836000

O -2.96078200 -0.95644000 0.58128800

H 1.62100300 -2.50907900 1.00154200

C -0.07742700 1.59140200 1.33437200

H -0.14193500 2.65421400 1.62256600

O 1.53989800 -1.57971400 0.77210100

H -1.29459700 -0.42898000 -1.05436000

O 0.69489600 0.84896500 1.88480600

H 1.45311100 -1.07468800 1.58930700

H -1.07023900 3.10674000 -0.74157000

H 0.12408600 1.99213500 -1.43253600

O -2.71697500 -0.71147300 -1.79427200

H -3.03282400 -0.91813000 -0.57139600

C -1.63966300 -1.18331500 0.79829700

C 3.00205300 -0.08444200 -0.81285600

O 3.94572200 -0.74344700 -0.75559600

O 2.08079800 0.61064500 -0.90285900

H 0.15601400 -0.54045800 -0.09777900

N -0.82946200 -0.25208400 -0.10659500

H -2.03751800 1.26490700 0.61348300

H -2.76705000 -1.51597800 -2.31725100

H -1.33795300 -0.97523200 1.82874500

H -1.29035300 -2.18411800 0.51089600

25

Pb^{w}

C 0.36835300 -0.58094900 1.57286200

C 1.28783300 -0.76346200 0.36146300

H 0.58526700 0.37596600 2.04884100

O 1.63117400 2.46965400 0.96235100

H -1.13142700 -0.81239600 -1.26810200

C 0.94679400 -2.09820200 -0.26470400

H 1.56025500 -2.94657300 0.09061900

O -1.71738300 -0.05118000 -1.14261000

H 2.84553900 -0.12566300 1.54670100

O 0.06094100 -2.27204800 -1.06769400

H -2.40602900 -0.33715700 -0.53638200

H -0.67587000 -0.58324300 1.26259300

H 0.53531600 -1.37668800 2.30287800

O 2.96026200 1.22579800 3.06621400

H 2.32946600 1.86578600 2.70986900

C 2.59058200 2.34889300 0.24079600

C -0.67734200 2.24658900 -0.47215600

O -0.00906400 2.25019300 -1.41642900

O -1.34516600 2.30127200 0.46813100

H 3.30828900 -0.57562200 0.02640300

N 2.67805600 -0.78801000 0.79144700

H 1.04987400 0.01465200 -0.37653800

H 2.58727800 0.88599000 3.88442100

H 2.47895400 2.34873200 -0.85324900

H 3.60405200 2.23602500 0.65428000
